# Supplementary material for: Effects of unconditional cash transfers on family processes and wellbeing among mothers with low incomes
Source: Nat Commun. 2025 Aug 13;16:7517. doi: 10.1038/s41467-025-62438-x (PMC12350725; doi:10.1038/s41467-025-62438-x)
Supplement: Supplementary file 1 — Supplementary Information [file 41467_2025_62438_MOESM1_ESM.pdf]

Supplementary Fig. 1 CONSORT diagrams for age 1, 2, and 3 data collection waves..........2

Supplementary Table 1 Baseline characteristics of age 1 sample (N = 931)..............................3

Supplementary Table 2 Baseline characteristics of age 2 sample (N = 922)..............................4

Supplementary Table 3 Baseline characteristics of age 3 sample (N = 922)..............................5

Supplementary Table 4 Preregistered outcomes, organized by primary journal articles related to family economic investments (Gennetian et al. 2024)[1], Family Stress Models (this article), or other papers..6

Supplementary Table 5 Summary of family well-being and family process measures 8

Supplementary Table 6 Summary of ITT estimates of the impacts of the BFY high-cash gift on family well-being and family processes measures with full scales and subscales. 12

Supplementary Table 7 Description of how measures were categorized into analytic families in this study compared with categorization in the BFY preregistration..15

Supplementary Table 8 Summary of ITT estimates of the impacts of the BFY high-cash gift on family well-being and family processes measures with p-value adjustments following the preregistration plan. 16

Supplementary Table 9 Summary of ITT estimates of the impacts of the BFY high-cash gift on family well-being and family processes measures constructed from common items across ages.19

Supplementary Table 10 Summary of ITT estimates of the impacts of the BFY high-cash gift on family well-being and family processes measures with analytic weights to improve baseline balance between the high- and low-cash gift groups. 20

Supplementary Table 11 Summary of ITT estimates of the impacts of the BFY high-cash gift on family well-being and family processes measures with analytic weights for non-response....23

Supplementary Table 12 Summary of ITT estimates of the impacts of the BFY high-cash gift on family well-being and family processes measures using multiple imputation to correct for missing data. 26

Supplementary Table 13 Summary of ITT estimates of impacts of the BFY high-cash gift on family well-being and family processes measures moderated by the presence of the child's biological father in the mother's household at birth. 28

Appendix Table 14 Summary of ITT estimates of impacts of the BFY high-cash gift on family well-being and family processes measures moderated by the mother's self-identified racial and ethnic background. 33

Appendix Table 15 Summary of ITT estimates of impacts of the BFY high-cash gift on family well-being and family processes measures moderated by reported high household income, measured as an income greater than sample median 38

Supplementary References 43

Supplementary Fig. 1 CONSORT diagrams for age 1, 2, and 3 data collection waves. Following Consolidated Standards of Reporting Trials (CONSORT), the figure reports the number of observations tracked across waves. **a** Wave 1 (child age 1) data collection (June 2019–June 2020). **b** Wave 2 (child age 2) data collection (July 2020–June 2021). **c** Wave 3 (child age 3) data collection (July 2021–June 2022).

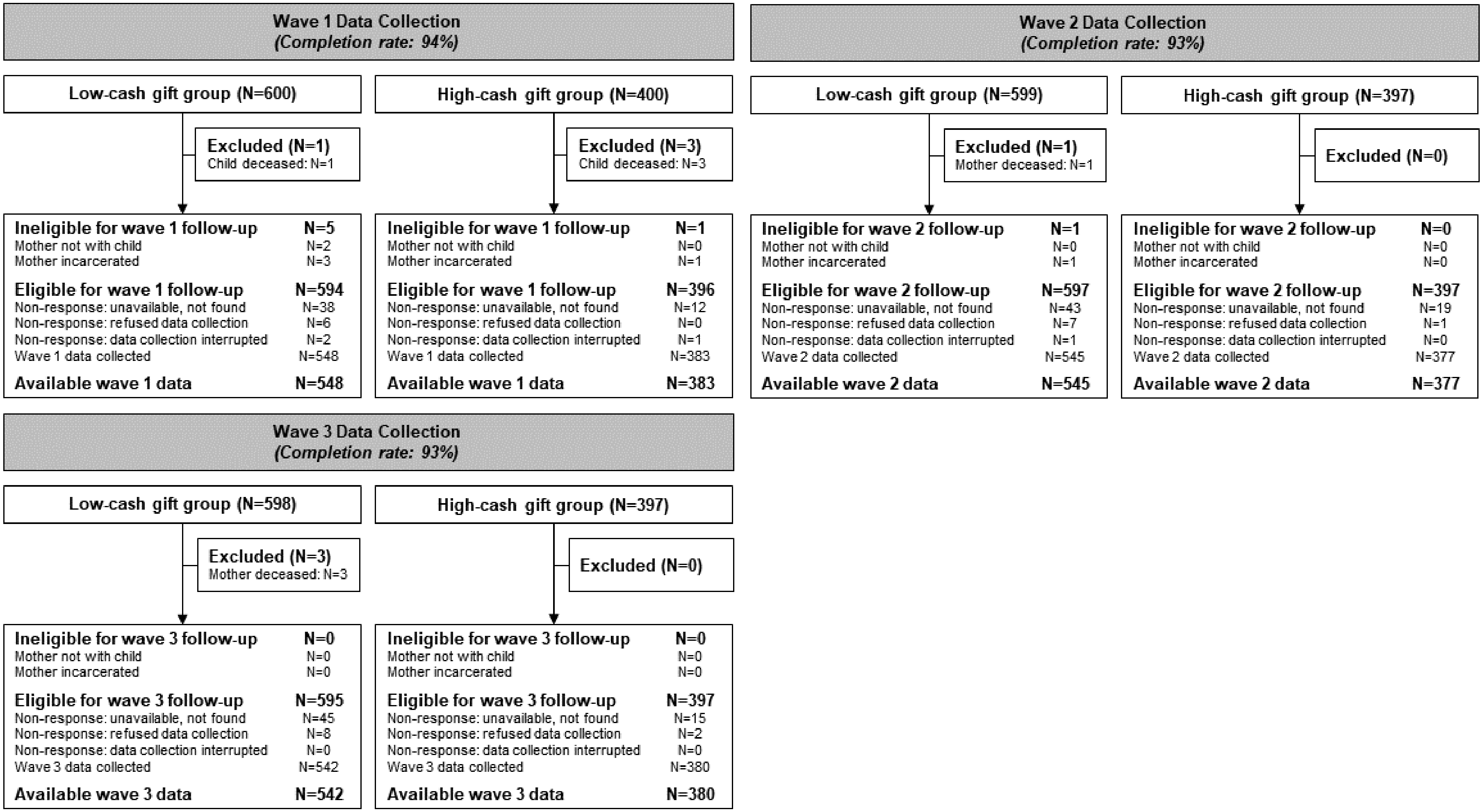

Supplementary Table 1 Baseline characteristics of age 1 sample ($N = 931$).

	Low-Cash Gift	High-Cash Gift	Std. Mean Difference		
	Mean (SD)	Mean (SD)	Hedges' g	Cox's Index	*p*-value
Child					
Female	.51	.48		-.07	.40
Weight at birth (lb)	7.14 (1.08)	7.11 (1.02)	-.02		.73
Gestational age (weeks)	39.09 (1.23)	39.03 (1.25)	-.05		.49
Mother					
Age at birth (years)	26.94 (5.84)	27.41 (5.75)	.08		.19
Education (years)	11.86 (2.83)	11.91 (2.98)	.02		.78
Race/ethnicity					
White, non-Hispanic	.11	.08		-.21	.16
Black, non-Hispanic	.39	.44		.12	.07
Multiple, non-Hispanic	.04	.03		-.18	.37
Other or unknown	.04	.02		-.43	.07
Hispanic	.42	.42		.00	.77
Marital status					
Never married	.42	.50		.20	.02
Single, living with partner	.27	.21		-.20	.05
Married	.22	.21		-.04	1.00
Divorced/separated	.05	.03		-.32	.18
Other or unknown	.05	.05		.00	.76
Health is good or better	.88	.92		.27	.02
Depression (CES-D)	.68 (.44)	.67 (.45)	-.01		.85
Cigarettes per week during pregnancy	4.68 (20.32)	3.11 (11.10)	-.09		.12
Alcohol drinks per week during pregnancy	.15 (1.66)	.03 (.39)	-.10		.09
Number of children born to mother	2.42 (1.37)	2.53 (1.42)	.08		.24
Number of adults in household	2.08 (.98)	2.02 (.97)	-.06		.35
Biological father in household	.41	.35		-.15	.06
Household income ($1,000s)	22.31 (21.28)	20.98 (16.01)	-.07		.30
Household income unknown	.06	.07		.10	.62
Household net worth ($1,000s)	-2.19 (29.37)	-3.27 (20.72)	-.04		.54
Household net worth unknown	.11	.11		.00	1.00
Joint test: $\chi^2(28)=29.54$, *p*-value=.39, *N*=927					

Notes: The *p*-values were derived from a series of two-sided OLS bivariate regressions in which each baseline characteristic was regressed on the treatment status indicator using robust standard errors and site-level fixed effects. Joint test of orthogonality was conducted using a probit model with robust standard errors and site-level fixed effects. Standardized mean differences were calculated using Hedges' g for continuous variables and Cox's Index for dichotomous variables. The number of observations with non-missing baseline measures ranges between 489 and 548 for the low-cash gift group and between 342 and 383 for the high-cash gift group. If more than 10 cases were missing for a covariate, missing data dummies were included in the table and the joint test. If fewer than 10 cases were missing, missing data dummies were not included in the table but were included in the joint test. Chi-square tests of independence were conducted for the two categorical variables: mother race/ethnicity and mother marital status. For both tests, $p > .05$. All respondents with missing data on gestational age are in the control group, so this dummy is excluded from the joint test due to perfect collinearity. CES-D=Center for Epidemiologic Studies Depression Scale.

Supplementary Table 2 Baseline characteristics of age 2 sample (N = 922).

	Low-Cash Gift	High-Cash Gift	Std. Mean Difference		
	Mean (SD)	Mean (SD)	Hedges' g	Cox's Index	*p*-value
Child					
Female	.50	.48		-.05	.43
Weight at birth (lb)	7.14 (1.07)	7.10 (1.03)	-.03		.63
Gestational age (weeks)	39.10 (1.25)	39.04 (1.19)	-.05		.44
Mother					
Age at birth (years)	26.90 (5.82)	27.45 (5.77)	.09		.15
Education (years)	11.96 (2.81)	11.92 (2.99)	-.02		.84
Race/ethnicity					
White, non-Hispanic	.10	.08		-.15	.24
Black, non-Hispanic	.40	.44		.10	.16
Multiple, non-Hispanic	.04	.03		-.18	.34
Other or unknown	.05	.02		-.57	.04
Hispanic	.41	.42		.02	.50
Marital status					
Never married	.42	.50		.20	.02
Single, living with partner	.26	.21		-.17	.08
Married	.22	.22		.00	.96
Divorced/separated	.05	.03		-.32	.18
Other or unknown	.05	.05		.00	.68
Health is good or better	.89	.93		.30	.04
Depression (CES-D)	.66 (.44)	.67 (.44)	.02		.75
Cigarettes per week during pregnancy	4.71 (20.33)	3.28 (11.42)	-.08		.15
Alcohol drinks per week during pregnancy	.15 (1.67)	.03 (.39)	-.10		.09
Number of children born to mother	2.40 (1.38)	2.53 (1.41)	.09		.19
Number of adults in household	2.09 (.98)	2.03 (.98)	-.06		.35
Biological father in household	.40	.34		-.16	.09
Household income ($1,000s)	22.04 (18.77)	20.91 (16.00)	-.06		.35
Household income unknown	.06	.07		.10	.44
Household net worth ($1,000s)	-1.81 (29.80)	-3.21 (20.84)	-.05		.43
Household net worth unknown	.11	.11		.00	.83
Joint test: $\chi^2(28)$=30.88, *p*-value=.32, *N*=918					

Notes: The *p*-values were derived from a series of two-sided OLS bivariate regressions in which each baseline characteristic was regressed on the treatment status indicator using robust standard errors and site-level fixed effects. Joint test of orthogonality was conducted using a probit model with robust standard errors and site-level fixed effects. Standardized mean differences were calculated using Hedges' g for continuous variables and Cox's Index for dichotomous variables. The number of observations with non-missing baseline measures ranges between 483 and 545 for the low-cash gift group and between 336 and 377 for the high-cash gift group. If more than 10 cases were missing for a covariate, missing data dummies were included in the table and the joint test. If fewer than 10 cases were missing, missing data dummies were not included in the table but were included in the joint test. Chi-square tests of independence were conducted for the two categorical variables: mother race/ethnicity and mother marital status. For both tests, $p > .05$. All respondents with missing data on gestational age are in the control group, so this dummy is excluded from the joint test due to perfect collinearity. CES-D=Center for Epidemiologic Studies Depression Scale.

Supplementary Table 3 Baseline characteristics of age 3 sample (N = 922).

	Low-Cash Gift	High-Cash Gift	Std. Mean Difference		
	Mean (SD)	Mean (SD)	Hedges' g	Cox's Index	*p*-value
Child					
Female	.50	.48		-.05	.49
Weight at birth (lb)	7.15 (1.06)	7.10 (1.01)	-.05		.48
Gestational age (weeks)	39.08 (1.27)	39.04 (1.24)	-.03		.67
Mother					
Age at birth (years)	26.89 (5.87)	27.36 (5.77)	.08		.20
Education (years)	11.98 (2.78)	11.87 (2.96)	-.04		.60
Race/ethnicity					
White, non-Hispanic	.11	.08		-.21	.10
Black, non-Hispanic	.39	.44		.12	.08
Multiple, non-Hispanic	.04	.03		-.18	.20
Other or unknown	.05	.02		-.57	.03
Hispanic	.41	.43		.05	.41
Marital status					
Never married	.42	.50		.20	.02
Single, living with partner	.26	.21		-.17	.07
Married	.21	.22		.04	.82
Divorced/separated	.05	.03		-.32	.18
Other or unknown	.06	.05		-.12	.42
Health is good or better	.88	.92		.27	.03
Depression (CES-D)	.68 (.46)	.68 (.45)	.00		.92
Cigarettes per week during pregnancy	5.03 (21.25)	3.26 (11.39)	-.10		.09
Alcohol drinks per week during pregnancy	.17 (1.70)	.03 (.39)	-.11		.05
Number of children born to mother	2.40 (1.39)	2.53 (1.41)	.09		.18
Number of adults in household	2.11 (.99)	2.03 (.98)	-.08		.23
Biological father in household	.40	.35		-.13	.09
Household income ($1,000s)	22.48 (21.90)	20.78 (15.89)	-.09		.19
Household income unknown	.06	.07		.10	.40
Household net worth ($1,000s)	-1.79 (29.91)	-2.24 (12.79)	-.02		.77
Household net worth unknown	.11	.11		.00	.78
Joint test: $\chi^2(28)$=34.17, *p*-value=.20, N=918					

Notes: The *p*-values were derived from a series of two-sided OLS bivariate regressions in which each baseline characteristic was regressed on the treatment status indicator using robust standard errors and site-level fixed effects. Joint test of orthogonality was conducted using a probit model with robust standard errors and site-level fixed effects. Standardized mean differences were calculated using Hedges' g for continuous variables and Cox's Index for dichotomous variables. The number of observations with non-missing baseline measures ranges between 480 and 543 for the low-cash gift group and between 339 and 383 for the high-cash gift group. If more than 10 cases were missing for a covariate, missing data dummies were included in the table and the joint test. If fewer than 10 cases were missing, missing data dummies were not included in the table but were included in the joint test. Chi-square tests of independence were conducted for the two categorical variables: mother race/ethnicity and mother marital status. For both tests, $p > .05$. All respondents with missing data on gestational age are in the control group, so this dummy is excluded from the joint test due to perfect collinearity. CES-D=Center for Epidemiologic Studies Depression Scale.

Supplementary Table 4 Preregistered outcomes, organized by primary journal articles related to family economic investments (Gennetian et al. 2024)[1], Family Stress Models (this article), or other papers.

	Family Investment	Family Stress	Other	Ages
Maternal and Family-Focused Preregistered Outcomes				
Household poverty status	S			1, 2, 3
Maternal global happiness	S			1, 2, 3
Maternal agency (HOPE scale)	S			1, 2, 3
Number of benefits (social services) received by mother	S			1, 2, 3
Mother's education and training participation and attainment	S			1, 2, 3
Index of child-focused expenditures (since birth)	S			1, 2, 3
Index of child-focused expenditures (in past 30 days)	S			1, 2, 3
Cost of paid childcare last week	S			1, 2, 3
Use of center-based care in last year	S			1
Use of center-based care in last week	S			2, 3
Parent-child activities index	S	S		1, 2, 3
Index of food insecurity		S		1, 2, 3
Index of economic stress		S		1, 2, 3
Maternal perceived stress (PSS)		S		1, 2, 3
Maternal parenting stress		S		1, 2
Physiological stress (maternal hair cortisol)		S		1
Maternal depression (PHQ-8)		S		1, 2, 3
Maternal anxiety (GAD-7)		S		2, 3
Maternal anxiety (Beck Anxiety Inventory)		S		1, 3
Physical abuse		S		1,2
Frequency of arguing		S		1,2
Romantic relationship quality		S		1, 2, 3
Spanking discipline strategy		S		1, 2, 3
Mother's positive parenting behaviors (PICCOLO)		S		
Mother's time to labor market reentry from birth			S	1
Mother's time to full-time labor market reentry from birth			S	1
Index of perceptions of neighborhood safety			S	1, 2, 3
Index of housing quality			S	1
Homelessness			S	1, 2, 3
Excessive residential mobility			S	1, 2, 3
Neighborhood poverty			S	1, 2, 3
Alcohol and cigarette use			S	1, 3
Opioid use			S	1, 3
Index of chaos in the home			S	1, 2
Maternal global health			S	1, 2

Maternal sleep	S	1, 3
Adult word count (LENA)	S	1
Conversational turns (LENA)	S	1
Child-Focused Preregistered Outcomes		
Maternal concern for language delay	P	1
Socioemotional problems (BITSEA)	S	1, 2
Behavior/emotional problems	P	3
Maternal concern for behavioral and social-emotional problems	P	3
Wave 1 resting brain function	S	1
Sleep problems[a]	P, S	1, 2, 3
Index of overall health[a]	P, S	1, 2, 3
Consumption of healthy foods	S	2
Consumption of unhealthy foods	S	2
Parents' Evaluation of Developmental Status (PEDS)	S	3
Total "predictive concerns" in the PEDS	S	3

[a]Registered as secondary outcome in ages 1 and 2, and as primary outcome in age 3. P=primary outcome, S=secondary outcome, PSS=Perceived Stress Scale, PHQ-8=Personal Health Questionnaire Depression scale. GAD-7=General Anxiety Disorder-7, BITSEA=Brief Infant-Toddler Social and Emotional Assessment, LENA= Language Environment Analysis, PICCOLO=Parenting Interaction with Children: Checklist of Observations Linked to Outcomes.

Supplementary Table 5 Summary of family well-being and family process measures

Outcome Measures	Number of Items			Item Lists	Cronbach's α			Source / Note
	Age 1	Age 2	Age 3		Age 1	Age 2	Age 3	
Panel 1: Economic Resources								
Income-to-needs ratio with gift	-	NA	NA	How much did you earn from all your employers before taxes and deductions during [previous year]?	-	NA	NA	Total household income divided by federal poverty level based on family sizes at each age
Household income with gift	-	NA	NA	How much did you earn from all your employers before taxes and deductions during [previous year]?	-	NA	NA	Total household income
Panel 2: Economic Pressure								
Food insecurity index[a] (range: 0–6)	5	6	6	In the last 12 months,<br>1) couldn't afford to eat balanced meals. *(often/sometimes, never true)*<br>2) the food that we bought didn't last, and we didn't have money to get more.<br>3) eat less than you felt you should because there wasn't enough money for food.<br>4) ever hungry, but didn't eat, because there wasn't enough money for food.[age2,age3]<br>5) cut the size of your meals or skip meals because there wasn't enough money for food.<br>6) cut the size of your meals or skip meals more than 3 months. *(yes, no)*	.86	.85	.87	Additive index of food insecurity (U.S. Department of Agriculture 2012)[2]
Non-food economic hardship index[a] (range: 0–5)	5	5	4	In the last 12 months, *(yes, no)*<br>1) missed a rent or mortgage payment?<br>2) miss a payment for oil, gas, water, or electricity?<br>3) forced to leave or were evicted from your home?<br>4) when you or your child needed medical or dental care but did not get it?<br>5) miss a payment for your phone, internet, cable or streaming services?[age1,age2]	.54	.57	.46	Additive index of select items in the economic stress index (Kling et al. 2007)[3]
Economic worry (range: 0–5)	1	1	1	How often do you worry about being able to meet your monthly living expenses? *(all the time, very frequently, occasionally, rarely, very rarely, never)*	NA	NA	NA	1 item from the economic stress index (Kling et al. 2007)[3]
Panel 3: Parent Psychological Distress								
Perceived stress index[a] (range: 0–40)	9	9	10	In the last months, how often have you *(never, almost never, sometimes, fairly often, very often)*	.75	.75	.79	Additive index (Cohen et al. 1994, 1983)[4,5]
Subindex: distress (range: 0–24)	6	6	6	1) been upset because of something that happened unexpectedly?<br>2) felt nervous and "stressed"?<br>3) found that you could not cope with all the things that you had to do?<br>4) been angered because of things that were outside of your control?<br>5) felt difficulties were piling up so high that you could not overcome them?<br>6) felt that you were unable to control the important things in your life?	.84	.86	.87	Additive subindex (Hewitt et al. 1992)[6]
Subindex: coping (range: 0–16)	3	3	4	1) felt confident about your ability to handle your personal problems?<br>2) been able to control irritations in your life?<br>3) felt that you were on top of things?<br>4) felt that things were going your way?[age3]	.64	.69	.78	Additive subindex (Hewitt et al. 1992)[6]
Parenting stress index (range: 7–35)	7	7	-	*(strongly disagree, disagree, not sure, agree, strongly agree)*	.55	.55	-	Additive index of two indices from two sources
Subindex: aggravation (range: 3–15)	3	3	-	1) I find myself giving up more of my life to meet my [child]'s needs than I ever expected.<br>2) I feel trapped by my responsibilities as a parent.<br>3) Since having children, I have been unable to do new and different things.	.55	.57	-	Panel Study of Income Dynamics–Child Development Supplement (PSID-CDS) (Schickedanz et al. 2018)[7]
Subindex: parenting competence (range: 4–20)	4	4	-	1) When it comes to raising kids, I have a lot of confidence in my abilities.<br>2) I feel good about my parenting ability.<br>3) I can admit my flaws as a parent, and still think I am a pretty good one.<br>4) I think my kids will grow up to say I was a wonderful parent.	.82	.79	-	Project GAIN (https://uwsc.wisc.edu/the-wisconsin-families-study-wiscfams/)

Maternal depression: PHQ-8 (range: 0–24)	8	8	8	In the past 2 weeks, how often have you *(not at all, several days, more than half the days, nearly every day)*<br>1) been bothered because you had little interest or pleasure in doing things?<br>2) been bothered from feeling down, depressed, or hopeless?<br>3) had trouble falling or staying asleep, or sleeping too much?<br>4) been bothered by feeling tired or had little energy?<br>5) been bothered by a poor appetite or overeating?<br>6) been bothered by feeling bad about yourself or that you are a failure or have let yourself or your family down?<br>7) been bothered from having trouble concentrating on things, such as reading the newspaper or watching television?<br>8) being bothered from moving or speaking so slowly that other people could have noticed. Or, the opposite being so fidgety or restless that you have been moving around a lot more than usual?	.84	.85	.87	Additive scale (Kroenke et al. 2009)[8]
Maternal anxiety: GAD-7 (range:0–21)	-	7	7	Over the last two weeks, (*not at all, several days, more than half the days, nearly every days*)<br>1) feeling nervous, anxious, or on edge.<br>2) not being able to stop or control worrying.<br>3) worrying too much about different things.<br>4) trouble relaxing.<br>5) being so restless that it is hard to sit still.<br>6) becoming easily annoyed or irritable.<br>7) feeling afraid, as if something awful might happen.	-	.90	.90	Spitzer et al. (2006)[9]
Maternal anxiety: Beck Anxiety Inventory (range: 0–63)	21	-	21	In the past month, I was bothered by (not at all, mildly, moderately, severely)	.90	-	.92	Additive scale (Beck et al. 1988)[10]
Subscale: somatic (range: 0–45)	15	-	15	1) numbness or tingling.<br>2) feeling hot.<br>3) wobbliness in legs.<br>4) feeling unable to relax.<br>5) feeling dizzy or lightheaded.<br>6) heart pounding or racing.<br>7) feeling unsteady.<br>8) a feeling of choking.<br>9) hands trembling.<br>10) feeling shaky or unsteady.<br>11) difficulty breathing.<br>12) indigestion.<br>13) feeling faint or lightheaded.<br>14) face flushed.<br>15) hot or cold sweats.	.84	-	.89	Subscale adaptation of Creamer et al. (1995)[11]
Subscale: psychological (range: 0–18)	6	-	6	1) fear of the worst happening.<br>2) feeling terrified or afraid.<br>3) feeling nervous.<br>4) fear of losing control.<br>5) fear of dying.<br>6) feeling scared.	.85	-	.85	Subscale adaptation of Creamer et al. (1995)[11]
Physiological Stress. Hair Cortisol	NA	-	-	Measure of cortisol from mother's hair sample	NA	-	-	Meyer et al. (2014)[12]
Panel 4: Interparental Relationship Quality								

Co-parenting quality (range: 7–21)	7	7	-	*(always, sometimes, or rarely true)*	.90	.88	-	Additive index (McLanahan and Beck 2010)[13]
Subindex: trust (range:4–12)	4	4	-	1) When [partner] is with [child], he acts like the kind of father you want for your child. Would you say it's always true, sometimes true, or rarely true?<br>2) You can trust him to take good care of [child].<br>3) You can count on [partner] for help when you need someone to look after [child] for a few hours.<br>4) If you had to go away for one week and could not take [child] with you, how much would you trust [partner] to take care of [child]? *(very much, somewhat, not at all)*	.83	.78	-	Authors' arrangement based on factor analysis
Subindex: cooperative (range:3–9)	3	3	-	1) He respects the schedules and rules you make for [child].<br>2) He supports you in the way you want to raise [child].<br>3) You and [partner] talk about problems that come up with [child].	.81	.78	-	Authors' arrangement based on factor analysis
Ever hit by partner (range: 0–1)	1	1	-	Ever cut, bruised, or seriously hurt, in a fight, with your current partner? *(yes/no)*	NA	NA	NA	1 item from Future of Families and Child Wellbeing Study
Argue with partner (range: 1–5)	1	1	-	How often do you and your current partner argue about the things that are important to you? *(always, often, sometimes, rarely, never)*	NA	NA	NA	1 item from Future of Families and Child Wellbeing Study
Relationship quality[a] (range: 11–33)	10	11	11	How often *(often, sometimes, never)*	.83	.75	.85	Additive scale adapted from Future of Families and Child Wellbeing Study
Subindex: supportive (range: 4–12)	4	4	4	1) was your partner fair and willing to compromise when you had a disagreement?<br>2) did your partner express affection or love for you?<br>3) did your partner encourage or help you to do things that were important to you?<br>4) did your partner listen to you when you needed someone to talk to?	.80	.73	.81	Additive subindex (Turney 2015)[14]
Subindex: emotional abuse (range: 2–6)	2	2	2	1) did your partner insult or criticize you or your ideas?<br>2) did your partner make you feel down or bad about yourself during an argument?	.80	.67	.81	Additive subindex (Turney 2015)[14]
Subindex: physical abuse (range: 5–15)	4	5	5	1) did your partner try to keep you from seeing or talking with your friends or family, or try to prevent you from going to work or school?<br>2) did your partner hit, slap, kick, or otherwise hurt you physically?<br>3) did your partner try to make you have sex or do sexual things you didn't want to do?<br>4) did your partner make you feel afraid?<br>5) has your partner ever threatened to spank or slap your child or children?[age2,age3]	.73	.59	.76	Additive subindex (Turney 2015)[14]
Panel 5: Parenting Quality								
Parent-child activities[a] (range: 5–20)	4	5	4	How often do you (*every day, few times a week, few times a month, rarely/not at all*)<br>1) read books or look at pictures in a book with [child]?<br>2) tell stories to [child]?<br>3) play together with toys for building things?<br>4) play pretend games?[age2,age3]<br>5) go to any out-of-the home activities or programs that are specifically for babies, like Mommy and Me, library story times, and play groups?[age1,age2]	.61	.66	.67	Additive index (Rodriguez and Tamis-LeMonda 2011)[15]
Parent-child interaction: PICCOLO (range: 0–58)	29	-	-	(*absent, barely, clearly*)<br>1) Speaks in a warm tone.<br>2) Smiles at child.<br>3) Praises child.<br>4) Is physically close to child.<br>5) Uses positive expressions with child.<br>6) Is engaged and interacting with child.<br>7) Shows emotional warmth.	.75	-	-	Observational assessment (Roggman et al. 2013)[16]

				8) Pays attention to what child is doing.<br>9) Changes pace or activity to meet child’s interests or needs.<br>10) Is flexible about child’s change of activities or interests.<br>11) Follows what child is trying to do.<br>12) Responds to child’s emotions.<br>13) Looks at child when child talks or makes sounds.<br>14) Replies to child’s words or sounds.<br>15) Waits for child’s response after making a suggestion.<br>16) Encourages child to handle toys.<br>17) Supports child in making choices.<br>18) Supports child in doing things on his or her own.<br>19) Verbally encourages child’s efforts.<br>20) Offers suggestions to help the child.<br>21) Shows enthusiasm about what child is doing.<br>22) Explains reasons for something to the child.<br>23) Suggests activities to extend what the child is doing.<br>24) Repeats or expands child’s words or sounds.<br>25) Labels objects or actions for the child.<br>26) Engages in pretend play with child.<br>27) Does activities in a sequence of steps.<br>28) Talks to child about characteristics of objects.<br>29) Asks child for information.				
Spanking as discipline (range: 0–1)	1	1	1	In the past month have you spanked your [age]-year-old child because they were misbehaving or acting up? *(yes/no)*	NA	NA	NA	1 binary item (Reichman et al. 2001)

[a]For five measures, the number of items that make up the index or scale are not identical across ages (sometimes missing due to survey error).

Notes: Data collection occurred in July 2019 to June 2020 for age 1, July 2020 to July 2021 for age 2, and July 2021 to July 2022 for age 3. Full references for the measures are provided in the Supplementary References. Household incomes across all years are inflation-adjusted to 2019 dollars, and the poverty line is based on the 2019 U.S. Census poverty threshold. Income-to-needs is the household income divided by the poverty line for a given family size and composition. Income and income-to-needs have been truncated at the 99th percentile. Superscripts (e.g., [age1,age2]) in the item lists indicate age-specific items. For example, [age2,age3] means that the item is asked only at ages 2 and 3. “-” indicates missing information. NA=not applicable. PHQ-8=Personal Health Questionnaire Depression scale. GAD-7=General Anxiety Disorder-7. PICCOLO=Parenting Interaction with Children: Checklist of Observations Linked to Outcomes. The range of scores provides the possible range based on scoring the items, not the observed range based on responses.

Supplementary Table 6 Summary of ITT estimates of the impacts of the BFY high-cash gift on family well-being and family processes measures with full scales and subscales.

Family	Outcome	Hypoth.		Age 1	Age 2	Age 3	Pooled Sample
Panel 3: Parent Psychological Distress							
3	Perceived stress index	-	Effect (con. interval)	.62 (-.18, 1.41)	.45 (-.34, 1.24)	.75 (-.14, 1.65)	.63 (-.02, 1.27)
			Std. effect	.10	.07	.10	.09
			N (deg. freedom)	930 (883)	920 (874)	921 (875)	2,771 (973)
			p-value	.13	.26	.10	.06
	Distress	-	Effect (con. interval)	.52 (-.14, 1.18)	.13 (-.51, .76)	.51 (-.16, 1.18)	.40 (-.11, .92)
			Std. effect	.10	.03	.09	.08
			N (deg. freedom)	930 (883)	919 (873)	920 (874)	2,769 (972)
			p-value	.12	.69	.14	.13
	Coping (reverse)	-	Effect (con. interval)	.11 (-.29, .50)	.35 (-.05, .76)	.24 (-.28, .75)	.24 (-.08, .56)
			Std. effect	.04	.12	.06	.07
			N (deg. freedom)	926 (879)	911 (865)	921 (875)	2,758 (971)
			p-value	.60	.09	.37	.15
3	Parenting stress index	-	Effect (con. interval)	.53 (.06, .99)	.52 (.06, .98)		.52 (.12, .92)
			Std. effect	.15	.15		.15
			N (deg. freedom)	929 (882)	918 (872)		1,847 (964)
			p-value	.03	.03		.01
	Aggravation	-	Effect (con. interval)	.41 (.06, .77)	.24 (-.11, .59)		.33 (.02, .63)
			Std. effect	.16	.09		.12
			N (deg. freedom)	924 (877)	915 (869)		1,839 (963)
			p-value	.02	.17		.03
	Parenting competence (reverse)	-	Effect (con. interval)	.11 (-.17, .39)	.28 (.01, .56)		.20 (-.03, .42)
			Std. effect	.06	.14		.10
			N (deg. freedom)	929 (882)	918 (872)		1,847 (964)
			p-value	.43	.04		.09
3	Maternal anxiety (Beck Anxiety Inventory)	-	Effect (con. interval)	1.66 (.66, 2.66)		-.04 (-1.02, .93)	.80 (-.05, 1.64)
			Std. effect	.25		-.01	.12
			N (deg. freedom)	930 (883)		919 (873)	1,849 (967)
			p-value	.00		.93	.06
	Somatic	-	Effect (con. interval)	.96 (.29, 1.63)		-.22 (-.90, .46)	.36 (-.21, .93)
			Std. effect	.21		-.04	.09
			N (deg. freedom)	930 (883)		919 (873)	1,849 (967)
			p-value	.00		.52	.22

	Psychological	-	Effect (con. interval)	.70 (.27, 1.13)		.18 (-.20, .55)	.44 (.10, .78)
			Std. effect	.25		.06	.16
			N (deg. freedom)	930 (883)		919 (873)	1,849 (967)
			p-value	.00		.35	.01
Panel 4: Interparental Relationship Quality							
4	Co-parenting relationship quality	+	Effect (con. interval)	-.38 (-.85, .09)	-.34 (-.80, .12)		-.34 (-.73, .05)
			Std. effect	-.13	-.12		-.12
			N (deg. freedom)	720 (673)	663 (617)		1,383 (802)
			p-value	.12	.15		.09
	Trust	+	Effect (con. interval)	-.17 (-.46, .11)	-.16 (-.42, .11)		-.16 (-.39, .07)
			Std. effect	-.10	-.10		-.09
			N (deg. freedom)	719 (672)	658 (612)		1,377 (801)
			p-value	.23	.25		.18
	Cooperative	+	Effect (con. interval)	-.17 (-.38, .05)	-.21 (-.43, .01)		-.18 (-.36, .00)
			Std. effect	-.13	-.16		-.14
			N (deg. freedom)	717 (670)	662 (616)		1,379 (802)
			p-value	.12	.07		.05
4	Romantic relationship quality index	+	Effect (con. interval)	-.30 (-.91, .32)	-.32 (-.81, .18)	-.62 (-1.18, -.06)	-.47 (-.86, -.08)
			Std. effect	-.08	-.12	-.17	-.14
			N (deg. freedom)	572 (525)	512 (467)	793 (747)	1,877 (900)
			p-value	.35	.21	.03	.02
	Supportive	+	Effect (con. interval)	-.40 (-.76, -.04)	-.11 (-.44, .21)	-.36 (-.68, -.03)	-.31 (-.55, -.08)
			Std. effect	-.20	-.07	-.17	-.16
			N (deg. freedom)	570 (523)	511 (466)	793 (747)	1,874 (899)
			p-value	.03	.49	.03	.01
	Emotional abuse (reverse)	+	Effect (con. interval)	.08 (-.11, .27)	-.09 (-.25, .07)	-.12 (-.29, .05)	-.06 (-.18, .06)
			Std. effect	.07	-.11	-.10	-.07
			N (deg. freedom)	571 (524)	511 (466)	792 (746)	1,874 (899)
			p-value	.42	.27	.17	.31
	Physical abuse (reverse)	+	Effect (con. interval)	.02 (-.19, .24)	-.11 (-.24, .02)	-.13 (-.31, .05)	-.10 (-.21, .02)
			Std. effect	.02	-.14	-.11	-.10
			N (deg. freedom)	570 (523)	511 (466)	793 (747)	1,874 (900)
			p-value	.83	.10	.16	.11

Note: Each block of rows presents, for each outcome, the raw treatment effect with confidence intervals in parentheses; the standardized treatment effect size; number of observations and degrees of freedom; and the *p*-values. The ITT estimates come from two-sided regressions with site fixed effects, controlling for baseline covariates, child age at interview, and phone interview status. Outcomes were standardized using the standard deviation of the low-cash gift within each age. We report the degrees of freedom computed as the sample size minus the number of parameters estimated in the model. This statistic is complicated in the pooled sample because we cluster the standard error to adjust for non-independence. For simplicity, we report the default degrees of freedom reported in most software,

which is the number of clusters minus one. The *p*-value comes from analyses that do not correct for multiple outcomes. The "Pooled Sample" column presents estimates from analyses that pool observations across ages, adjust for age indicators, and cluster the standard error at the individual level. Preregistered, hypothesized directions of the intervention effects are presented with "+" or "-" for directional increase or decrease in the outcome, respectively. The direction of the subindices and subscales match the main index or scale. For example, the positively oriented subindex "parenting competence" has been recoded such that higher values reflect lower parenting competence (i.e., more stress).

Supplementary Table 7 Description of how measures were categorized into analytic families in this study compared with categorization in the BFY preregistration.

Outcome	Not Preregistered	Paper Arrangement of Outcomes	Preregistered Arrangement of Outcomes
Income-to-needs ratio with gift	X	1. Economic Resources	1. Household Economic Hardship
Household income with gift	X	1. Economic Resources	Not Preregistered
Food insecurity index		2. Economic Pressure	1. Household Economic Hardship
Non-food economic hardship	X	2. Economic Pressure	1. Household Economic Hardship
Expense worry	X	2. Economic Pressure	1. Household Economic Hardship
Perceived stress index		3. Parent Psychological Distress	2. Family and Maternal Perceived Stress
Parenting stress index		3. Parent Psychological Distress	2. Family and Maternal Perceived Stress
Maternal depression (PHQ-8)		3. Parent Psychological Distress	3. Maternal Mental Health
Maternal anxiety (GAD-7)		3. Parent Psychological Distress	3. Maternal Mental Health
Maternal anxiety (Beck Anxiety Inventory)		3. Parent Psychological Distress	3. Maternal Mental Health
Physiological stress (ln hair cortisol)		3. Parent Psychological Distress	4. Maternal Physiological Stress
Co-parent relationship quality	X	4a. Interparental Relationship Quality	5. Co-Parent Quality
Romantic relationship quality index		4a. Interparental Relationship Quality	6. Maternal Relationship
Ever cut/bruised/seriously hurt by partner		4a. Interparental Relationship Quality	6. Maternal Relationship
Frequency of arguing		4a. Interparental Relationship Quality	6. Maternal Relationship
Parent-child activities index		4b. Parenting Quality	7. Frequency of Parent-Child Interaction
Parent-child interaction (PICCOLO)		4b. Parenting Quality	8. Parent-Child Interaction
Spanking discipline strategy		4b. Parenting Quality	9. Maternal Discipline

Notes: The table lists all the outcome measures examined in this paper and a few additional measures not in the paper, and indicates which measures were preregistered. Outcome measures are arranged into families of outcomes to implement the preregistered Westfall and Young's (1993)[18] step-down resampling methods of addressing multiple hypothesis testing. Thus, the arrangement of outcomes into families create different adjustments to the p-values. The third column shows the arrangement of outcome measures into families that correspond to the conceptual model used in the paper, the authors' adaptation of the family stress model (Masarik and Conger 2017)[19]. The fourth column shows the arrangement of outcomes original preregistration plans. PHQ-8=Personal Health Questionnaire Depression Scale. GAD-7=General Anxiety Disorder-7. PICCOLO=Parenting Interactions with Children: Checklist of Observations Linked to Outcomes.

Supplementary Table 8 Summary of ITT estimates of the impacts of the BFY high-cash gift on family well-being and family processes measures with *p*-value adjustments following the preregistration plan.

Family	Outcome	Hypoth.		Age 1	Age 2	Age 3	Pooled Sample
Panel 1: Economic Resources							
1	Income-to-needs ratio with gift	+	Effect (con. interval)		.10 (.00, .20)	.11 (.00, .22)	.11 (.02, .19)
			Std. effect		.12	.13	.13
			N (deg. freedom)		922 (876)	922 (876)	1,844 (956)
			p-value (WY *p*-value)		.04 (.17)	.04 (.16)	.01 (.05)
1	Household income with gift ($1,000s, in 2019 dollars)	+	Effect (con. interval)		2.79 (-.09, 5.68)	2.76 (-.32, 5.83)	2.86 (.37, 5.35)
			Std. effect		.11	.11	.11
			N (deg. freedom)		922 (876)	922 (876)	1,844 (956)
			p-value (WY *p*-value)		.06 (.20)	.08 (.26)	.03 (.09)
Panel 2: Economic Pressure							
1	Food insecurity index	-	Effect (con. interval)	.23 (-.00, .46)	-.00 (-.24, .23)	.05 (-.17, .27)	.10 (-.08, .28)
			Std. effect	.14	-.00	.03	.06
			N (deg. freedom)	929 (882)	921 (875)	920 (874)	2,770 (972)
			p-value (WY *p*-value)	.05 (.14)	.98 (.98)	.68 (.89)	.27 (.44)
1	Non-food economic hardship index	-	Effect (con. interval)	.04 (-.12, .20)	.07 (-.09, .23)	.02 (-.09, .14)	.05 (-.06, .16)
			Std. effect	.04	.06	.03	.04
			N (deg. freedom)	930 (883)	921 (875)	922 (876)	2,773 (972)
			p-value (WY *p*-value)	.62 (.62)	.38 (.72)	.68 (.89)	.41 (.44)
1	Expense worry	-	Effect (con. interval)	.17 (-.04, .38)	.08 (-.14, .29)	.11 (-.10, .31)	.12 (-.04, .28)
			Std. effect	.10	.05	.06	.07
			N (deg. freedom)	930 (883)	919 (873)	919 (873)	2,768 (972)
			p-value (WY *p*-value)	.12 (.22)	.48 (.72)	.31 (.64)	.14 (.33)
Panel 3: Parent Psychological Distress							
2	Perceived stress index	-	Effect (con. interval)	.62 (-.18, 1.41)	.45 (-.34, 1.24)	.75 (-.14, 1.65)	.63 (-.02, 1.27)
			Std. effect	.10	.07	.10	.09
			N (deg. freedom)	930 (883)	920 (874)	921 (875)	2,771 (973)
			p-value (WY *p*-value)	.13 (.13)	.26 (.26)	.10 (.10)	.06 (.06)
2	Parenting stress index	-	Effect (con. interval)	.53 (.06, .99)	.52 (.06, .98)		.52 (.12, .92)
			Std. effect	.15	.15		.15
			N (deg. freedom)	929 (882)	918 (872)		1,847 (964)
			p-value (WY *p*-value)	.03 (.05)	.03 (.05)		.01 (.02)
3	Maternal depression (PHQ-8)	-	Effect (con. interval)	.26 (-.29, .80)	.33 (-.20, .86)	-.03 (-.56, .50)	.19 (-.22, .60)
			Std. effect	.06	.08	-.01	.05
			N (deg. freedom)	930 (883)	919 (873)	919 (873)	2,768 (973)
			p-value (WY *p*-value)	.35 (.35)	.22 (.32)	.91 (.99)	.37 (.39)

3	Maternal anxiety (GAD-7)	-	Effect (con. interval)		.30 (-.22, .82)	.17 (-.34, .69)	.25 (-.19, .68)
			Std. effect		.08	.04	.06
			N (deg. freedom)		919 (873)	921 (875)	1,840 (956)
			p-value (WY *p*-value)		.26 (.32)	.51 (.81)	.27 (.39)
3	Maternal anxiety (Beck Anxiety Inventory)	-	Effect (con. interval)	1.66 (.66, 2.66)		-.04 (-1.02, .93)	.80 (-.05, 1.64)
			Std. effect	.25		-.01	.12
			N (deg. freedom)	930 (883)		919 (873)	1,849 (967)
			p-value (WY *p*-value)	.00 (.00)		.93 (.99)	.06 (.14)
4	Physiological stress (ln hair cortisol)	-	Effect (con. interval)	.03 (-.26, .32)			.03 (-.26, .32)
			Std. effect	.02			.02
			N (deg. freedom)	364 (317)			364 (363)
			p-value (WY *p*-value)	.84 (.84)			.84 (.84)
Panel 4: Interparental Relationship Quality							
5	Co-parenting relationship quality	+	Effect (con. interval)	-.38 (-.85, .09)	-.34 (-.80, .12)		-.34 (-.73, .05)
			Std. effect	-.13	-.12		-.12
			N (deg. freedom)	720 (673)	663 (617)		1,383 (802)
			p-value (WY *p*-value)	.12 (.11)	.15 (.15)		.09 (.09)
6	Romantic relationship quality index	+	Effect (con. interval)	-.30 (-.91, .32)	-.32 (-.81, .18)	-.62 (-1.18, -.06)	-.47 (-.86, -.08)
			Std. effect	-.08	-.12	-.17	-.14
			N (deg. freedom)	572 (525)	512 (467)	793 (747)	1,877 (900)
			p-value (WY *p*-value)	.34 (.69)	.21 (.39)	.03 (.03)	.02 (.06)
6	Ever cut/bruised/seriously hurt by partner	-	Effect (con. interval)	-.02 (-.07, .02)	.01 (-.01, .04)		-.00 (-.03, .02)
			Std. effect	-.08	.12		.02
			N (deg. freedom)	572 (525)	511 (466)		1,083 (770)
			p-value (WY *p*-value)	.35 (.69)	.33 (.39)		.74 (.74)
6	Frequency of arguing	-	Effect (con. interval)	-.04 (-.21, .14)	.11 (-.04, .27)		.05 (-.07, .17)
			Std. effect	-.04	.13		.06
			N (deg. freedom)	566 (519)	512 (467)		1,078 (766)
			p-value (WY *p*-value)	.67 (.69)	.15 (.39)		.43 (.68)
Panel 5: Parenting Quality							
7	Parent-child activities index	+	Effect (con. interval)	.44 (.09, .79)	.43 (.05, .81)	.38 (.05, .72)	.42 (.14, .70)
			Std. effect	.16	.14	.15	.15
			N (deg. freedom)	929 (882)	919 (873)	915 (869)	2,763 (971)
			p-value (WY *p*-value)	.01 (.01)	.03 (.03)	.02 (.02)	.00 (.00)
8	Parent-child interaction (PICCOLO)	+	Effect (con. interval)	.53 (-.42, 1.48)			.53 (-.42, 1.48)
			Std. effect	.10			.10
			N (deg. freedom)	543 (496)			543 (542)

			p-value (WY *p*-value)	.28 (.27)			.28 (.27)
9	Spanking discipline strategy	+	Effect (con. interval)	.02 (-.02, .06)	-.05 (-.10, -.01)	-.03 (-.08, .02)	-.03 (-.06, .01)
			Std. effect	.08	-.14	-.07	-.06
			N (deg. freedom)	596 (549)	914 (868)	917 (871)	2,427 (959)
			p-value (WY *p*-value)	.40 (.40)	.02 (.02)	.27 (.27)	.14 (.14)

Notes: Each block of rows presents, for each outcome, the raw treatment effect with confidence intervals in parentheses; the standardized treatment effect size; number of observations and degrees of freedom; and the *p*-values and Westfall and Young (WY) (1993)[18] adjusted *p*-values. The ITT estimates come from two-sided regressions with site fixed effects, controlling for baseline covariates, child age at interview, and phone interview status. Outcomes were standardized using the standard deviation of the low-cash gift within each age. We report the degrees of freedom computed as the sample size minus the number of parameters estimated in the model. This statistic is complicated in the pooled sample because we cluster the standard error to adjust for non-independence. For simplicity, we report the default degrees of freedom reported in most software, which is the number of clusters minus one. The *p*-value comes from analyses that do not correct for multiple outcomes, while WY *p*-value is based on Westfall and Young's (1993)[18] step-down resampling methods of addressing multiple hypothesis testing, where outcomes are grouped in families (following Fig. 1) and their *p*-values adjusted within each family. The "Pooled Sample" column presents estimates from analyses that pool observations across ages, adjust for age indicators, and cluster the standard error at the individual level. Preregistered, hypothesized directions of the intervention effects are presented with "+" or "-" for directional increase or decrease in the outcome, respectively. Household incomes across all years are inflation-adjusted to 2019 dollars, and the poverty line is based on the 2019 U.S. Census poverty threshold. Income-to-needs is the household income divided by the poverty line for a given family size and composition. Income and income-to-needs have been truncated at the 99th percentile. PHQ-8=Personal Health Questionnaire Depression scale. GAD-7=General Anxiety Disorder-7. PICCOLO=Parenting Interaction with Children: Checklist of Observations Linked to Outcomes.

Supplementary Table 9 Summary of ITT estimates of the impacts of the BFY high-cash gift on family well-being and family processes measures constructed from common items across ages.

Family	Outcome	Hypoth.		Age 1	Age 2	Age 3	Pooled Sample
Panel 2: Economic Pressure							
2	Food insecurity index	-	Effect (con. interval)	.23 (-.00, .46)	.00 (-.20, .21)	.07 (-.13, .26)	.11 (-.06, .27)
			Std. effect	.14	.00	.04	.07
			N (deg. freedom)	929 (882)	919 (873)	919 (873)	2,767 (972)
			p-value	.05	.97	.51	.20
2	Non-food economic hardship index	-	Effect (con. interval)	.04 (-.07, .16)	.06 (-.06, .18)	.02 (-.09, .14)	.04 (-.04, .13)
			Std. effect	.05	.07	.03	.05
			N (deg. freedom)	929 (882)	920 (874)	922 (876)	2,771 (972)
			p-value	.47	.31	.68	.32
Panel 3: Maternal Psychological Distress							
3	Perceived stress index	-	Effect (con. interval)	.62 (-.18, 1.41)	.45 (-.34, 1.24)	.69 (-.12, 1.51)	.60 (-.02, 1.23)
			Std. effect	.10	.07	.10	.09
			N (deg. freedom)	930 (883)	920 (874)	921 (875)	2,771 (973)
			p-value	.13	.26	.10	.06
Panel 4: Interparental Relationship Quality							
4	Romantic relationship quality index	+	Effect	-.30 (-.91, .32)	-.28 (-.76, .21)	-.62 (-1.17, -.08)	-.46 (-.85, -.07)
			Std. effect	-.08	-.11	-.17	-.14
			N (deg. freedom)	572 (525)	512 (467)	793 (747)	1,877 (900)
			p-value	.35	.26	.03	.02
Panel 5: Parenting Quality							
5	Parent-child activities index	+	Effect (con. interval)	.40 (.09, .71)	.31 (.05, .58)	.33 (.06, .59)	.35 (.13, .57)
			Std. effect	.17	.15	.17	.16
			N (deg. freedom)	929 (882)	918 (872)	914 (868)	2,761 (971)
			p-value	.01	.02	.01	.00

Notes: For measures with missing items in some ages, this table shows the estimate on measures constructed using only items that appear in all the ages. Each block of rows presents, for each outcome, the raw treatment effect with confidence intervals in parentheses; the standardized treatment effect size; number of observations and degrees of freedom; and the *p*-values. The ITT estimates come from two-sided regressions with site fixed effects, controlling for baseline covariates, child age at interview, and phone interview status. Outcomes were standardized using the standard deviation of the low-cash gift within each age. We report the degrees of freedom computed as the sample size minus the number of parameters estimated in the model. This statistic is complicated in the pooled sample because we cluster the standard error to adjust for non-independence. For simplicity, we report the default degrees of freedom reported in most software, which is the number of clusters minus one. The *p*-value comes from analyses that do not correct for multiple outcomes. The "Pooled Sample" column presents estimates from analyses that pool observations across ages, adjust for age indicators, and cluster the standard error at the individual level. Preregistered, hypothesized directions of the intervention effects are presented with "+" or "-" for directional increase or decrease in the outcome, respectively.

Supplementary Table 10 Summary of ITT estimates of the impacts of the BFY high-cash gift on family well-being and family processes measures with analytic weights to improve baseline balance between the high- and low-cash gift groups.

Family	Outcome	Hypoth.		Age1	Age 2	Age 3	Pooled Sample
Panel 1: Economic Resources							
1	Income-to-needs ratio with gift	+	Effect (con. interval)		.11 (.01, .21)	.12 (-.01, .25)	.12 (.03, .21)
			Std. effect		.13	.14	.14
			N (deg. freedom)		922 (876)	922 (876)	1,844 (956)
			p-value		.03	.07	.01
1	Household income with gift ($1,000s, in 2019 dollars)	+	Effect (con. interval)		3.20 (.20, 6.19)	3.08 (-.44, 6.60)	3.23 (.55, 5.92)
			Std. effect		.13	.12	.13
			N (deg. freedom)		922 (876)	922 (876)	1,844 (956)
			p-value		.04	.09	.02
Panel 2: Economic Pressure							
2	Food insecurity index	-	Effect (con. interval)	.25 (-.01, .52)	.05 (-.20, .30)	.04 (-.19, .28)	.12 (-.07, .31)
			Std. effect	.15	.03	.03	.07
			N (deg. freedom)	929 (882)	921 (875)	920 (874)	2,770 (972)
			p-value	.06	.70	.70	.22
2	Non-food economic hardship index	-	Effect (con. interval)	.03 (-.16, .22)	.09 (-.08, .27)	.03 (-.10, .15)	.05 (-.07, .18)
			Std. effect	.02	.08	.03	.05
			N (deg. freedom)	930 (883)	921 (875)	922 (876)	2,773 (972)
			p-value	.79	.29	.66	.40
2	Expense worry	-	Effect (con. interval)	.14 (-.09, .37)	.08 (-.15, .31)	.12 (-.10, .34)	.12 (-.06, .29)
			Std. effect	.09	.05	.08	.07
			N (deg. freedom)	930 (883)	919 (873)	919 (873)	2,768 (972)
			p-value	.23	.49	.28	.18
Panel 3: Maternal Psychological Distress							
3	Perceived stress index	-	Effect (con. interval)	.21 (-.73, 1.16)	.40 (-.45, 1.24)	.76 (-.19, 1.71)	.50 (-.23, 1.22)
			Std. effect	.03	.06	.10	.07
			N (deg. freedom)	930 (883)	920 (874)	921 (875)	2,771 (973)
			p-value	.66	.36	.12	.18
3	Parenting stress index	-	Effect (con. interval)	.50 (-.01, 1.02)	.51 (.02, .99)		.51 (.09, .93)
			Std. effect	.14	.14		.14
			N (deg. freedom)	929 (882)	918 (872)		1,847 (964)
			p-value	.05	.04		.02
3	Maternal depression (PHQ-8)	-	Effect (con. interval)	.23 (-.41, .87)	.38 (-.21, .96)	-.08 (-.69, .53)	.19 (-.29, .66)
			Std. effect	.06	.10	-.02	.05
			N (deg. freedom)	930 (883)	919 (873)	919 (873)	2,768 (973)
			p-value	.48	.20	.79	.44

3	Maternal anxiety (GAD-7)	-	Effect (con. interval)		.30 (-.28, .87)	.20 (-.37, .77)	.26 (-.23, .75)
			Std. effect		.08	.05	.06
			N (deg. freedom)		919 (873)	921 (875)	1,840 (956)
			p-value		.32	.49	.29
3	Maternal anxiety (Beck Anxiety Inventory)	-	Effect (con. interval)	1.28 (.14, 2.42)		.12 (-.89, 1.14)	.70 (-.22, 1.62)
			Std. effect	.19		.02	.10
			N (deg. freedom)	930 (883)		919 (873)	1,849 (967)
			p-value	.03		.81	.14
3	Physiological stress (ln hair cortisol)	-	Effect (con. interval)	.15 (-.18, .49)			.15 (-.18, .49)
			Std. effect	.11			.11
			N (deg. freedom)	364 (317)			364 (363)
			p-value	.37			.37
Panel 4: Interparental Relationship Quality							
4	Co-parenting relationship quality	+	Effect (con. interval)	-.41 (-.94, .11)	-.40 (-.88, .09)		-.40 (-.82, .02)
			Std. effect	-.14	-.14		-.14
			N (deg. freedom)	720 (673)	663 (617)		1,383 (802)
			p-value	.12	.11		.06
4	Romantic relationship quality index	+	Effect (con. interval)	-.28 (-1.07, .50)	-.31 (-.79, .18)	-.88 (-1.43, -.34)	-.56 (-.97, -.15)
			Std. effect	-.08	-.11	-.24	-.16
			N (deg. freedom)	572 (525)	512 (467)	793 (747)	1,877 (900)
			p-value	.48	.22	.00	.01
4	Ever cut/bruised/seriously hurt by partner	-	Effect (con. interval)	-.02 (-.08, .04)	.01 (-.01, .04)		-.00 (-.03, .03)
			Std. effect	-.07	.11		.03
			N (deg. freedom)	572 (525)	511 (466)		1,083 (770)
			p-value	.50	.33		.91
4	Frequency of arguing	-	Effect (con. interval)	-.01 (-.19, .17)	.10 (-.06, .27)		.06 (-.07, .19)
			Std. effect	-.01	.13		.07
			N (deg. freedom)	566 (519)	512 (467)		1,078 (766)
			p-value	.89	.22		.40
Panel 5: Parenting Quality							
5	Parent-child activities index	+	Effect (con. interval)	.61 (.20, 1.01)	.47 (.06, .89)	.45 (.10, .81)	.51 (.19, .82)
			Std. effect	.23	.16	.18	.19
			N (deg. freedom)	929 (882)	919 (873)	915 (869)	2,763 (971)
			p-value	.00	.03	.01	.00
5	Parent-child interaction (PICCOLO)	+	Effect (con. interval)	.55 (-.48, 1.57)			.55 (-.48, 1.57)
			Std. effect	.10			.10
			N (deg. freedom)	543 (496)			543 (542)

			p-value	.30			.30
5	Spanking discipline strategy	+	Effect (con. interval)	.02 (-.02, .07)	-.05 (-.10, .00)	-.03 (-.09, .02)	-.03 (-.06, .01)
			Std. effect	.10	-.12	-.08	-.05
			N (deg. freedom)	596 (549)	914 (868)	917 (871)	2,427 (959)
			p-value	.26	.06	.27	.21

Notes: Each block of rows presents, for each outcome, the raw treatment effect with confidence intervals in parentheses; the standardized treatment effect size; number of observations and degrees of freedom; and the *p*-values. The estimates are conducted with the low-cash gift subsample being weighted by the odds of being in the high-cash gift group to look like the high-cash gift sample, on average. The estimates come from two-sided regressions with site fixed effects, controlling for baseline covariates, child age at interview, and phone interview status. We report the degrees of freedom computed as the sample size minus the number of parameters estimated in the model. This statistic is complicated in the pooled sample because we cluster the standard error to adjust for non-independence. For simplicity, we report the default degrees of freedom reported in most software, which is the number of clusters minus one. The number of observations is computed without weights. Outcomes were standardized using the standard deviation of the low-cash gift within each age. The *p*-value comes from analyses that do not correct for multiple outcomes. The "Pooled Sample" column presents estimates from analyses that pool observations across ages, adjust for age indicators, and cluster the standard error at the individual level. Preregistered, hypothesized directions of the intervention effects are presented with "+" or "-" for directional increase or decrease in the outcome, respectively. Household incomes across all years are inflation-adjusted to 2019 dollars, and the poverty line is based on the 2019 U.S. Census poverty threshold. Income-to-needs is the household income divided by the poverty line for a given family size and composition. Income and income-to-needs have been truncated at the 99th percentile. PHQ-8=Personal Health Questionnaire Depression scale. GAD-7=General Anxiety Disorder-7. PICCOLO=Parenting Interaction with Children: Checklist of Observations Linked to Outcomes.

Supplementary Table 11 Summary of ITT estimates of the impacts of the BFY high-cash gift on family well-being and family processes measures with analytic weights for non-response.

Family	Outcome	Hypoth.		Age 1	Age 2	Age 3	Pooled Sample
Panel 1: Economic Resources							
1	Income-to-needs ratio with gift	+	Effect (con. interval)		.10 (.00, .20)	.11 (.00, .22)	.11 (.02, .19)
			Std. effect		.12	.13	.13
			N (deg. freedom)		922 (876)	922 (876)	1,844 (956)
			p-value		.05	.04	.01
1	Household income with gift ($1,000s, in 2019 dollars)	+	Effect (con. interval)		2.80 (-.09, 5.70)	2.77 (-.31, 5.84)	2.87 (.37, 5.36)
			Std. effect		.11	.11	.11
			N (deg. freedom)		922 (876)	922 (876)	1,844 (956)
			p-value		.06	.08	.02
Panel 2: Economic Pressure							
2	Food insecurity index	-	Effect (con. interval)	.23 (-.00, .47)	-.01 (-.24, .22)	.04 (-.18, .26)	.10 (-.08, .27)
			Std. effect	.14	-.01	.02	.06
			N (deg. freedom)	929 (882)	921 (875)	920 (874)	2,770 (972)
			p-value	.05	.93	.74	.30
2	Non-food economic hardship index	-	Effect (con. interval)	.04 (-.12, .19)	.07 (-.09, .22)	.02 (-.10, .14)	.04 (-.07, .15)
			Std. effect	.03	.06	.02	.04
			N (deg. freedom)	930 (883)	921 (875)	922 (876)	2,773 (972)
			p-value	.66	.41	.75	.46
2	Expense worry	-	Effect (con. interval)	.17 (-.04, .38)	.07 (-.14, .29)	.10 (-.11, .30)	.12 (-.05, .28)
			Std. effect	.10	.05	.06	.07
			N (deg. freedom)	930 (883)	919 (873)	919 (873)	2,768 (972)
			p-value	.12	.50	.36	.16
Panel 3: Parent Psychological Distress							
3	Perceived stress index	-	Effect (con. interval)	.61 (-.19, 1.40)	.46 (-.33, 1.24)	.72 (-.18, 1.61)	.61 (-.03, 1.25)
			Std. effect	.10	.07	.10	.09
			N (deg. freedom)	930 (883)	920 (874)	921 (875)	2,771 (973)
			p-value	.14	.25	.12	.06
3	Parenting stress index	-	Effect (con. interval)	.53 (.06, 1.00)	.52 (.06, .99)		.53 (.12, .93)
			Std. effect	.15	.15		.15
			N (deg. freedom)	929 (882)	918 (872)		1,847 (964)
			p-value	.03	.03		.01
3	Maternal depression (PHQ-8)	-	Effect (con. interval)	.25 (-.29, .80)	.33 (-.20, .86)	-.06 (-.60, .47)	.18 (-.24, .59)
			Std. effect	.06	.08	-.01	.04
			N (deg. freedom)	930 (883)	919 (873)	919 (873)	2,768 (973)
			p-value	.36	.22	.81	.40

3	Maternal anxiety (GAD-7)	-	Effect (con. interval)		.29 (-.23, .81)	.15 (-.37, .67)	.23 (-.21, .66)
			Std. effect		.08	.03	.06
			N (deg. freedom)		919 (873)	921 (875)	1,840 (956)
			p-value		.27	.58	.30
3	Maternal anxiety (Beck Anxiety Inventory)	-	Effect (con. interval)	1.66 (.66, 2.65)		-.07 (-1.05, .90)	.77 (-.07, 1.61)
			Std. effect	.25		-.01	.12
			N (deg. freedom)	930 (883)		919 (873)	1,849 (967)
			p-value	.00		.88	.07
3	Physiological stress (ln hair cortisol)	-	Effect (con. interval)	.03 (-.26, .32)			.03 (-.26, .32)
			Std. effect	.02			.02
			N (deg. freedom)	364 (317)			364 (363)
			p-value	.82			.82
Panel 4: Interparental Relationship Quality							
4	Co-parenting quality	+	Effect (con. interval)	-.37 (-.84, .10)	-.36 (-.82, .11)		-.35 (-.74, .05)
			Std. effect	-.13	-.13		-.12
			N (deg. freedom)	720 (673)	663 (617)		1,383 (802)
			p-value	.12	.14		.09
4	Relationship quality index	+	Effect (con. interval)	-.29 (-.90, .33)	-.30 (-.80, .19)	-.61 (-1.17, -.05)	-.46 (-.85, -.07)
			Std. effect	-.08	-.11	-.17	-.14
			N (deg. freedom)	572 (525)	512 (467)	793 (747)	1,877 (900)
			p-value	.36	.23	.03	.02
4	Ever cut/bruised/seriously hurt by partner	-	Effect (con. interval)	-.02 (-.07, .02)	.01 (-.01, .04)		-.00 (-.03, .02)
			Std. effect	-.08	.11		.02
			N (deg. freedom)	572 (525)	511 (466)		1,083 (770)
			p-value	.34	.34		.74
4	Frequency of arguing	-	Effect (con. interval)	-.04 (-.21, .14)	.11 (-.05, .26)		.05 (-.07, .17)
			Std. effect	-.04	.13		.06
			N (deg. freedom)	566 (519)	512 (467)		1,078 (766)
			p-value	.68	.17		.44
Panel 5: Parenting Quality							
5	Parent-child activities index	+	Effect (con. interval)	.45 (.09, .80)	.43 (.05, .82)	.38 (.04, .71)	.42 (.14, .70)
			Std. effect	.17	.15	.15	.15
			N (deg. freedom)	929 (882)	919 (873)	915 (869)	2,763 (971)
			p-value	.01	.03	.03	.00
5	Parent-child interaction (PICCOLO)	+	Effect (con. interval)	.54 (-.42, 1.50)			.54 (-.42, 1.50)
			Std. effect	.10			.10
			N (deg. freedom)	543 (496)			543 (542)

			p-value	.27			.27
5	Spanking discipline strategy	+	Effect (con. interval)	.02 (-.02, .06)	-.05 (-.10, -.01)	-.03 (-.08, .02)	-.03 (-.06, .01)
			Std. effect	.09	-.14	-.08	-.06
			N (deg. freedom)	596 (549)	914 (868)	917 (871)	2,427 (959)
			p-value	.33	.02	.23	.14

Notes: Each block of rows presents, for each outcome, the raw treatment effect with confidence intervals in parentheses; the standardized treatment effect size; number of observations and degrees of freedom; and the *p*-values. The estimates are weighted using non-response weights, where the sample for each age was weighted by the inverse probability of having each sample look like the full study sample of 1,000 observations. The estimates come from two-sided regressions with site fixed effects, controlling for baseline covariates, child age at interview, and phone interview status. We report the degrees of freedom computed as the sample size minus the number of parameters estimated in the model. This statistic is complicated in the pooled sample because we cluster the standard error to adjust for non-independence. For simplicity, we report the default degrees of freedom reported in most software, which is the number of clusters minus one. The number of observations is computed without weights. Outcomes were standardized using the standard deviation of the low-cash gift within each age. The *p*-value comes from analyses that do not correct for multiple outcomes. The “Pooled Sample” column presents estimates from analyses that pool observations across ages, adjust for age indicators, and cluster the standard error at the individual level. Preregistered, hypothesized directions of the intervention effects are presented with “+” or “-” for directional increase or decrease in the outcome, respectively. Household incomes across all years are inflation-adjusted to 2019 dollars, and the poverty line is based on the 2019 U.S. Census poverty threshold. Income-to-needs is the household income divided by the poverty line for a given family size and composition. Income and income-to-needs have been truncated at the 99th percentile. PHQ-8=Personal Health Questionnaire Depression scale. GAD-7=General Anxiety Disorder-7. PICCOLO=Parenting Interaction with Children: Checklist of Observations Linked to Outcomes.

Supplementary Table 12 Summary of ITT estimates of the impacts of the BFY high-cash gift on family well-being and family processes measures using multiple imputation to correct for missing data.

Family	Outcome	Hypoth.		Age 1	Age 2	Age 3	Pooled Sample
Panel 1: Economic Resources							
			Effect (con. interval)		.10 (.00, .19)	.10 (-.01, .21)	.10 (.02, .19)
1	Income-to-needs ratio with gift	+	*N* (deg. freedom)		1,000 (953)	1,000 (953)	2,000 (999)
			p-value		.05	.06	.02
	Household income with gift		Effect (con. interval)		2.73 (-.13, 5.59)	2.50 (-.59, 5.59)	2.69 (.21, 5.18)
1	($1,000s, in 2019 dollars)	+	*N* (deg. freedom)		1,000 (953)	1,000 (953)	2,000 (999)
			p-value		.06	.11	.03
Panel 2: Economic Pressure							
			Effect (con. interval)	.22 (-.02, .45)	.02 (-.22, .26)	.08 (-.14, .31)	.11 (-.07, .29)
2	Food insecurity index	-	*N* (deg. freedom)	1,000 (952)	1,000 (953)	1,000 (953)	3,000 (999)
			p-value	.07	.87	.48	.22
	Non-food economic hardship		Effect (con. interval)	.04 (-.11, .20)	.08 (-.08, .24)	.03 (-.08, .15)	.05 (-.06, .17)
2	index	-	*N* (deg. freedom)	1,000 (952)	1,000 (953)	1,000 (953)	3,000 (999)
			p-value	.58	.30	.57	.33
			Effect (con. interval)	.14 (-.07, .36)	.09 (-.13, .30)	.12 (-.08, .33)	.12 (-.04, .28)
2	Expense worry	-	*N* (deg. freedom)	1,000 (952)	1,000 (953)	1,000 (953)	3,000 (999)
			p-value	.18	.43	.23	.13
Panel 3: Parent Psychological Distress							
			Effect (con. interval)	.51 (-.28, 1.31)	.44 (-.33, 1.21)	.76 (-.14, 1.66)	.60 (-.04, 1.23)
3	Perceived stress index	-	*N* (deg. freedom)	1,000 (952)	1,000 (953)	1,000 (953)	3,000 (999)
			p-value	.21	.26	.10	.07
			Effect (con. interval)	.50 (.04, .95)	.53 (.06, .99)		.52 (.12, .91)
3	Parenting stress index	-	*N* (deg. freedom)	1,000 (952)	1,000 (953)		2,000 (999)
			p-value	.03	.03		.01
			Effect (con. interval)	.23 (-.30, .77)	.33 (-.19, .85)	-.03 (-.56, .50)	.18 (-.23, .59)
3	Maternal depression (PHQ-8)	-	*N* (deg. freedom)	1,000 (952)	1,000 (953)	1,000 (953)	3,000 (999)
			p-value	.39	.22	.90	.39
			Effect (con. interval)		.30 (-.21, .81)	.19 (-.33, .72)	.25 (-.18, .69)
3	Maternal anxiety (GAD-7)	-	*N* (deg. freedom)		1,000 (953)	1,000 (953)	2,000 (999)
			p-value		.24	.47	.25
			Effect (con. interval)	1.57 (.60, 2.55)		.00 (-1.00, 1.01)	.77 (-.08, 1.62)
3	Maternal anxiety (Beck Anxiety Inventory)	-	*N* (deg. freedom)	1,000 (952)		1,000 (953)	2,000 (999)
			p-value	.00		.99	.08

3	Physiological stress (ln hair cortisol)	-	Effect (con. interval)<br>*N* (deg. freedom)<br>*p*-value	.01 (-.26, .28)<br>674 (627)<br>.92			.01 (-.26, .28)<br>674 (673)<br>.92
Panel 4: Interparental Relationship Quality							
4	Co-parenting quality	+	Effect (con. interval)<br>*N* (deg. freedom)<br>*p*-value	-.39 (-.88, .09)<br>790 (742)<br>.11	-.35 (-.81, .11)<br>745 (698)<br>.14		-.35 (-.75, .05)<br>1,535 (860)<br>.08
4	Relationship quality index	+	Effect (con. interval)<br>*N* (deg. freedom)<br>*p*-value	-.31 (-.95, .32)<br>674 (627)<br>.34	-.31 (-.79, .17)<br>590 (544)<br>.20	-.59 (-1.15, -.04)<br>879 (832)<br>.04	-.40 (-.80, -.00)<br>2,143 (974)<br>.05
4	Ever cut/bruised/seriously hurt by partner	-	Effect (con. interval)<br>*N* (deg. freedom)<br>*p*-value	-.02 (-.07, .02)<br>674 (627)<br>.35	.01 (-.01, .04)<br>590 (544)<br>.33		-.01 (-.03, .02)<br>1,264 (877)<br>.69
4	Frequency of arguing	-	Effect (con. interval)<br>*N* (deg. freedom)<br>*p*-value	-.04 (-.21, .13)<br>674 (627)<br>.68	.10 (-.05, .25)<br>590 (544)<br>.19		.04 (-.08, .16)<br>1,264 (877)<br>.52
Panel 5: Parenting Quality							
5	Parent-child activities index	+	Effect (con. interval)<br>*N* (deg. freedom)<br>*p*-value	.41 (.07, .76)<br>1,000 (952)<br>.02	.41 (.02, .80)<br>1,000 (953)<br>.04	.36 (.03, .69)<br>1,000 (953)<br>.03	.39 (.11, .67)<br>3,000 (999)<br>.01
5	Parent-child interaction (PICCOLO)	+	Effect (con. interval)<br>*N* (deg. freedom)<br>*p*-value	.44 (-.56, 1.44)<br>674 (627)<br>.39			.44 (-.56, 1.44)<br>674 (673)<br>.39
5	Spanking discipline strategy	+	Effect (con. interval)<br>*N* (deg. freedom)<br>*p*-value	.02 (-.03, .06)<br>674 (627)<br>.45	-.05 (-.10, -.01)<br>1,000 (953)<br>.03	-.03 (-.08, .02)<br>1,000 (953)<br>.27	-.03 (-.06, .01)<br>2,674 (999)<br>.14

Notes: Each block of rows presents, for each outcome, the raw treatment effect with confidence intervals in parentheses; number of observations and degrees of freedom; and the *p*-values. The estimates are calculated with multiple imputation by chained equations, or MICE, using linear regression and predictive mean matching and imputing 20 datasets. For some outcomes, the multiple imputation only imputes missing values within the valid sample, which indicates the sample that is supposed to answer the question for the outcome. For example, the relationship quality is supposed to ask a participant who has a partner. The ITT estimates come from two-sided regressions with site fixed effects, controlling for baseline covariates, child age at interview, and phone interview status. We report the degrees of freedom computed as the sample size minus the number of parameters estimated in the model. This statistic is complicated in the pooled sample because we cluster the standard error to adjust for non-independence. The *p*-value comes from analyses that do not correct for multiple outcomes. For simplicity, we report the default degrees of freedom reported in most software, which is the number of clusters minus one. The "Pooled Sample" column presents estimates from analyses that pool observations across ages, adjust for age indicators, and cluster the standard error at the individual level. Preregistered, hypothesized directions of the intervention effects are presented with "+" or "-" for directional increase or decrease in the outcome, respectively. Household incomes across all years are inflation-adjusted to 2019 dollars, and the poverty line is based on the 2019 U.S. Census poverty threshold. Income-to-needs is the household income divided by the poverty line for a given family size and composition. Income and income-to-needs have been truncated at the 99th percentile. PHQ-8=Personal Health Questionnaire Depression scale. GAD-7=General Anxiety Disorder-7. PICCOLO=Parenting Interaction with Children: Checklist of Observations Linked to Outcomes.

Supplementary Table 13 Summary of ITT estimates of impacts of the BFY high-cash gift on family well-being and family processes measures moderated by the presence of the child's biological father in the mother's household at birth.

Family	Outcome .	Var.		Age 1	Age 2	Age 3	Pooled Sample
Panel 1: Economic Resources							
1	Income-to-needs ratio (including the BFY gift)	High-cash gift	Effect (con. interval)		.07 (-.04, .18)	.16 (.03, .30)	.12 (.02, .22)
			Std. effect		.08	.19	.14
			p-value		.21	.02	.02
		High-cash X Father in HH	Effect (con. interval)		.08 (-.14, .29)	-.14 (-.36, .08)	-.03 (-.21, .15)
			Std. effect		.09	-.16	-.04
			p-value		.48	.21	.73
		Father in HH	Effect (con. interval)		.08 (-.07, .23)	.09 (-.07, .25)	.08 (-.04, .21)
			Std. effect		.09	.10	.10
			p-value		.31	.26	.20
			N (deg. freedom)		922 (875)	922 (875)	1844 (956)
1	Household income with gift ($1000s, in 2019 dollars)	High-cash gift	Effect (con. interval)		2.21 (-1.06, 5.47)	4.70 (.95, 8.45)	3.54 (.56, 6.52)
			Std. effect		.09	.18	.14
			p-value		.18	.01	.02
		High-cash X Father in HH	Effect (con. interval)		1.57 (-4.78, 7.91)	-5.22 (-11.64, 1.20)	-1.83 (-7.24, 3.57)
			Std. effect		.06	-.20	-.07
			p-value		.63	.11	.51
		Father in HH	Effect (con. interval)		2.01 (-2.60, 6.63)	2.86 (-1.92, 7.64)	2.40 (-1.51, 6.31)
			Std. effect		.08	.11	.09
			p-value		.39	.24	.23
			N (deg. freedom)		922 (875)	922 (875)	1844 (956)
Panel 2: Economic Pressure							
2	Food insecurity index	High-cash gift	Effect (con. interval)	.27 (-.02, .56)	.05 (-.24, .33)	.14 (-.13, .41)	.16 (-.06, .37)
			Std. effect	.16	.03	.08	.09
			p-value	.07	.76	.32	.15
		High-cash X Father in HH	Effect (con. interval)	-.11 (-.59, .37)	-.13 (-.63, .37)	-.24 (-.74, .26)	-.15 (-.54, .24)
			Std. effect	-.07	-.07	-.14	-.09
			p-value	.66	.61	.34	.45
		Father in HH	Effect (con. interval)	.11 (-.23, .46)	.03 (-.31, .37)	.36 (-.01, .73)	.16 (-.12, .44)
			Std. effect	.07	.02	.20	.09
			p-value	.51	.86	.06	.26
			N (deg. freedom)	929 (881)	921 (874)	920 (873)	2770 (972)
2	Non-food economic hardship Index	High-cash gift	Effect (con. interval)	.10 (-.10, .29)	.04 (-.15, .24)	-.02 (-.17, .12)	.04 (-.10, .18)
			Std. effect	.09	.04	-.02	.03
			p-value	.32	.66	.76	.55
		High-cash X Father in HH	Effect (con. interval)	-.15 (-.48, .17)	.07 (-.26, .41)	.13 (-.13, .38)	.02 (-.22, .25)
			Std. effect	-.13	.06	.14	.02

			p-value	.36	.67	.33	.90
		Father in HH	Effect (con. interval)	.08 (-.15, .31)	.04 (-.18, .26)	.00 (-.18, .18)	.04 (-.12, .20)
			Std. effect	.07	.04	.00	.04
			p-value	.48	.73	.96	.60
			N (deg. freedom)	930 (882)	921 (874)	922 (875)	2773 (972)
2	Expense worry	High-cash gift	Effect (con. interval)	.19 (-.09, .46)	.10 (-.17, .37)	-.02 (-.28, .25)	.09 (-.11, .30)
			Std. effect	.11	.06	-.01	.06
			p-value	.18	.46	.90	.38
		High-cash X Father in HH	Effect (con. interval)	-.05 (-.48, .38)	-.07 (-.51, .37)	.33 (-.10, .75)	.08 (-.26, .41)
			Std. effect	-.03	-.04	.20	.05
			p-value	.82	.76	.13	.65
		Father in HH	Effect (con. interval)	.01 (-.31, .34)	.06 (-.26, .38)	.05 (-.27, .38)	.04 (-.20, .28)
			Std. effect	.01	.04	.03	.02
			p-value	.95	.72	.74	.75
			N (deg. freedom)	930 (882)	919 (872)	919 (872)	2768 (972)
Panel 3: Maternal Psychological Distress							
3	Perceived stress Index	High-cash gift	Effect (con. interval)	.80 (-.19, 1.80)	.25 (-.75, 1.24)	.65 (-.52, 1.81)	.58 (-.25, 1.41)
			Std. effect	.13	.04	.09	.09
			p-value	.11	.62	.27	.17
		High-cash X Father in HH	Effect (con. interval)	-.49 (-2.14, 1.15)	.54 (-1.06, 2.15)	.28 (-1.58, 2.14)	.11 (-1.22, 1.44)
			Std. effect	-.08	.09	.04	.02
			p-value	.56	.51	.77	.87
		Father in HH	Effect (con. interval)	.42 (-.77, 1.62)	-.34 (-1.51, .84)	-.42 (-1.83, .99)	-.11 (-1.09, .87)
			Std. effect	.07	-.05	-.06	-.01
			p-value	.49	.57	.56	.82
			N (deg. freedom)	930 (882)	920 (873)	921 (874)	2771 (973)
3	Parenting stress index	High-cash gift	Effect (con. interval)	.77 (.20, 1.34)	.68 (.09, 1.27)		.72 (.23, 1.22)
			Std. effect	.22	.19		.20
			p-value	.01	.02		.00
		High-cash X Father in HH	Effect (con. interval)	-.65 (-1.60, .30)	-.42 (-1.36, .52)		-.53 (-1.34, .28)
			Std. effect	-.18	-.12		-.15
			p-value	.18	.38		.20
		Father in HH	Effect (con. interval)	.29 (-.43, 1.00)	.19 (-.56, .94)		.23 (-.40, .87)
			Std. effect	.08	.05		.07
			p-value	.43	.62		.47
			N (deg. freedom)	929 (881)	918 (871)		1847 (964)
3	Maternal depression (PHQ-8)	High-cash gift	Effect (con. interval)	.32 (-.39, 1.02)	.24 (-.43, .90)	-.18 (-.85, .50)	.13 (-.39, .66)
			Std. effect	.08	.06	-.04	.04
			p-value	.38	.48	.61	.61
		High-cash X Father in HH	Effect (con. interval)	-.16 (-1.29, .98)	.24 (-.89, 1.36)	.39 (-.72, 1.50)	.14 (-.75, 1.03)
			Std. effect	-.04	.06	.08	.03

			p-value	.79	.68	.49	.75
			Effect (con. interval)	.54 (-.29, 1.37)	.33 (-.38, 1.04)	.01 (-.81, .82)	.30 (-.29, .90)
		Father in HH	Std. effect	.13	.08	.00	.07
			p-value	.20	.37	.99	.32
			N (deg. freedom)	930 (882)	919 (872)	919 (872)	2768 (973)
3	Maternal anxiety (GAD-7)		Effect (con. interval)		.29 (-.37, .95)	-.01 (-.64, .62)	.15 (-.38, .68)
		High-cash gift	Std. effect		.08	-.00	.04
			p-value		.39	.98	.58
			Effect (con. interval)		.03 (-1.06, 1.12)	.49 (-.63, 1.61)	.26 (-.67, 1.19)
		High-cash X Father in HH	Std. effect		.01	.11	.06
			p-value		.95	.39	.58
			Effect (con. interval)		.21 (-.52, .95)	.14 (-.66, .95)	.18 (-.46, .83)
		Father in HH	Std. effect		.06	.03	.05
			p-value		.57	.73	.57
			N (deg. freedom)		919 (872)	921 (874)	1840 (956)
3	Maternal anxiety (Beck Anxiety Inventory)		Effect (con. interval)	1.69 (.40, 2.98)		-.51 (-1.70, .67)	.58 (-.46, 1.61)
		High-cash gift	Std. effect	.26		-.06	.10
			p-value	.01		.40	.28
			Effect (con. interval)	-.09 (-2.19, 2.01)		1.26 (-.86, 3.37)	.58 (-1.22, 2.39)
		High-cash X Father in HH	Std. effect	-.01		.15	.07
			p-value	.93		.24	.53
			Effect (con. interval)	.94 (-.41, 2.29)		-.57 (-2.11, .97)	.18 (-.99, 1.36)
		Father in HH	Std. effect	.14		-.07	.04
			p-value	.17		.47	.76
			N (deg. freedom)	930 (882)		919 (872)	1849 (967)
3	Physiological stress (Ln hair cortisol)		Effect (con. interval)	-.02 (-.41, .38)			-.02 (-.41, .38)
		High-cash gift	Std. effect	-.01			-.01
			p-value	.93			.93
			Effect (con. interval)	.13 (-.44, .70)			.13 (-.44, .70)
		High-cash X Father in HH	Std. effect	.09			.09
			p-value	.66			.66
			Effect (con. interval)	-.08 (-.50, .35)			-.08 (-.50, .35)
		Father in HH	Std. effect	-.06			-.06
			p-value	.73			.73
			N (deg. freedom)	364 (316)			364 (316)
Panel 4: Interparental Relationship Quality							
4	Co-parenting relationship quality		Effect (con. interval)	-.60 (-1.33, .12)	-.46 (-1.10, .19)		-.52 (-1.08, .04)
		High-cash gift	Std. effect	-.21	-.17		-.18
			p-value	.10	.16		.07
		High-cash X Father in HH	Effect (con. interval)	.48 (-.43, 1.40)	.26 (-.64, 1.15)		.38 (-.39, 1.15)
			Std. effect	.17	.09		.13

			p-value	.30	.58		.33
		Father in HH	Effect (con. interval)	.58 (-.06, 1.21)	.29 (-.31, .88)		.44 (-.05, .94)
			Std. effect	.20	.10		.16
			p-value	.07	.34		.08
			N (deg. freedom)	720 (672)	663 (616)		1383 (802)
4	Romantic relationship quality index	High-cash gift	Effect (con. interval)	-.43 (-1.22, .36)	-.15 (-.86, .55)	-.89 (-1.56, -.23)	-.61 (-1.10, -.13)
			Std. effect	-.12	-.06	-.24	-.18
			p-value	.29	.67	.01	.01
		High-cash X Father in HH	Effect (con. interval)	.35 (-.95, 1.64)	-.31 (-1.33, .72)	.67 (-.53, 1.87)	.33 (-.49, 1.15)
			Std. effect	.10	-.11	.18	.08
			p-value	.60	.56	.28	.43
		Father in HH	Effect (con. interval)	.05 (-.88, .97)	-.32 (-1.00, .36)	-.47 (-1.23, .29)	-.32 (-.86, .22)
			Std. effect	.01	-.12	-.13	-.10
			p-value	.92	.36	.22	.24
			N (deg. freedom)	572 (524)	512 (466)	793 (746)	1877 (900)
4	Ever cut/bruised/ seriously hurt by partner	High-cash gift	Effect (con. interval)	-.03 (-.09, .02)	.02 (-.02, .05)		-.01 (-.05, .02)
			Std. effect	-.12	.13		-.01
			p-value	.24	.42		.49
		High-cash X Father in HH	Effect (con. interval)	.03 (-.07, .13)	-.00 (-.06, .05)		.02 (-.04, .08)
			Std. effect	.11	-.03		.05
			p-value	.53	.89		.53
		Father in HH	Effect (con. interval)	.00 (-.07, .07)	-.00 (-.04, .03)		.00 (-.03, .04)
			Std. effect	.00	-.01		.01
			p-value	.97	.97		.86
			N (deg. freedom)	572 (524)	511 (465)		1083 (770)
4	Frequency of arguing	High-cash gift	Effect (con. interval)	-.06 (-.29, .17)	-.00 (-.24, .24)		-.02 (-.20, .15)
			Std. effect	-.06	-.00		-.02
			p-value	.61	.99		.82
		High-cash X Father in HH	Effect (con. interval)	.06 (-.28, .40)	.21 (-.11, .54)		.15 (-.10, .40)
			Std. effect	.06	.26		.17
			p-value	.73	.20		.23
		Father in HH	Effect (con. interval)	.14 (-.12, .40)	.03 (-.19, .25)		.07 (-.11, .25)
			Std. effect	.14	.04		.07
			p-value	.29	.77		.45
			N (deg. freedom)	566 (518)	512 (466)		1078 (766)
Panel 5: Parenting Quality							
5	Parent-child activities index	High-cash gift	Effect (con. interval)	.38 (-.07, .82)	.27 (-.21, .76)	.20 (-.21, .61)	.28 (-.07, .64)
			Std. effect	.14	.09	.08	.10
			p-value	.09	.27	.34	.11
		High-cash X Father in HH	Effect (con. interval)	.16 (-.56, .89)	.42 (-.36, 1.19)	.49 (-.18, 1.16)	.36 (-.22, .93)
			Std. effect	.06	.14	.20	.13

			p-value	.66	.29	.15	.22
		Father in HH	Effect (con. interval)	-.13 (-.67, .42)	.07 (-.55, .69)	.26 (-.28, .79)	.07 (-.39, .53)
			Std. effect	-.05	.02	.10	.03
			p-value	.65	.81	.35	.77
			N (deg. freedom)	929 (881)	919 (872)	915 (868)	2763 (971)
5	Parent-child interaction (PICCOLO)	High-cash gift	Effect (con. interval)	.77 (-.45, 1.99)			.77 (-.45, 1.99)
			Std. effect	.14			.14
			p-value	.22			.22
		High-cash X Father in HH	Effect (con. interval)	-.68 (-2.46, 1.11)			-.68 (-2.46, 1.11)
			Std. effect	-.12			-.12
			p-value	.46			.46
		Father in HH	Effect (con. interval)	.96 (-.39, 2.31)			.96 (-.39, 2.31)
			Std. effect	.18			.18
			p-value	.16			.16
			N (deg. freedom)	543 (495)			543 (542)
5	Spanking discipline strategy	High-cash gift	Effect (con. interval)	.01 (-.04, .06)	-.04 (-.10, .02)	-.00 (-.07, .07)	-.01 (-.06, .03)
			Std. effect	.04	-.10	-.00	-.02
			p-value	.71	.23	.99	.64
		High-cash X Father in HH	Effect (con. interval)	.02 (-.05, .10)	-.05 (-.14, .05)	-.08 (-.18, .02)	-.04 (-.11, .03)
			Std. effect	.09	-.12	-.19	-.10
			p-value	.57	.35	.13	.23
		Father in HH	Effect (con. interval)	-.05 (-.10, .01)	-.03 (-.10, .05)	.01 (-.07, .09)	-.02 (-.07, .03)
			Std. effect	-.20	-.07	.02	-.06
			p-value	.09	.49	.86	.52
			N (deg. freedom)	596 (548)	914 (867)	917 (870)	2427 (959)

Notes: Data collection occurred in July 2019 to June 2020 for age 1, July 2020 to July 2021 for age 2, and July 2021 to July 2022 for age 3. Each block of rows presents for each outcome three coefficients from the same regression model: 1) high-cash gift group (treatment status); 2) an interaction term between high-gift group assignment and the presence of the child's biological fathers in the mother's household at the time of random assignment, and 3) the presence of the child's biological fathers in the mother's household at the time of random assignment. For all coefficients the raw coefficient in provided with confidence intervals in parentheses; the standardized coefficient effect size; and the *p*-value. The last row of each outcome presents number of observations and degrees of freedom. Covariates in all models include site fixed-effects, baseline covariates, child age at interview, and phone interview status. The "Pooled Sample" column presents estimates from analyses that pool observations across ages, adjust for age indicators, and cluster the standard error at the individual level. We report the degrees of freedom which is computed as the sample size minus the number of parameters estimated in the model. This statistic is complicated in the pooled sample because we cluster the standard error to adjust for non-independence. For simplicity, we report the default degrees of freedom reported in most software, which is the number of clusters minus one. The *p*-value comes from two-sided tests that do not correct for multiple outcomes. Household incomes across all years are inflation-adjusted to 2019 dollars, and the poverty line is based on the 2019 U.S. Census poverty threshold. Income-to-needs is the household income divided by the poverty line for a given family size and composition. Income and income-to-needs have been truncated at the 99th percentile. PHQ-8=Personal Health Questionnaire Depression scale. GAD-7=General Anxiety Disorder-7. PICCOLO=Parenting Interaction with Children: Checklist of Observations Linked to Outcomes.

Appendix Table 14 Summary of ITT estimates of impacts of the BFY high-cash gift on family well-being and family processes measures moderated by the mother's self-identified racial and ethnic background**.**

Family	Outcome	Var.		Age 1	Age 2	Age 3	Pooled Sample
Panel 1: Economic Resources							
1	Income-to-needs ratio (including the BFY gift)	High-cash gift	Effect (con. interval)		.08 (-.06, .22)	.07 (-.08, .23)	.08 (-.04, .20)
			Std. effect		.10	.09	.10
			p-value		.24	.36	.17
		High-cash X Mother Hispanic	Effect (con. interval)		.01 (-.18, .21)	.04 (-.19, .27)	.02 (-.16, .20)
			Std. effect		.02	.04	.03
			p-value		.88	.75	.81
		Mother Hispanic	Effect (con. interval)		.18 (.00, .35)	.14 (-.04, .33)	.17 (.03, .30)
			Std. effect		.21	.17	.19
			p-value		.04	.12	.02
			N (deg. freedom)		922 (877)	922 (877)	1844 (956)
1	Household Income with gift ($1000s, in 2019 dollars)	High-cash gift	Effect (con. interval)		3.02 (-1.07, 7.11)	2.05 (-2.46, 6.56)	2.69 (-.79, 6.16)
			Std. effect		.12	.08	.11
			p-value		.15	.37	.13
		High-cash X Mother Hispanic	Effect (con. interval)		-.58 (-6.50, 5.34)	.92 (-5.74, 7.58)	.04 (-5.26, 5.34)
			Std. effect		-.02	.04	.00
			p-value		.85	.79	.99
		Mother Hispanic	Effect (con. interval)		7.00 (1.78, 12.21)	6.11 (.57, 11.65)	6.69 (2.44, 10.95)
			Std. effect		.28	.24	.26
			p-value		.01	.03	.00
			N (deg. freedom)		922 (877)	922 (877)	1844 (956)
Panel 2: Economic Pressure							
2	Food Insecurity Index	High-cash gift	Effect (con. interval)	.22 (-.14, .57)	-.09 (-.46, .28)	-.14 (-.47, .19)	.01 (-.26, .28)
			Std. effect	.13	-.05	-.08	.01
			p-value	.24	.64	.40	.96
		High-cash X Mother Hispanic	Effect (con. interval)	.14 (-.35, .62)	.42 (-.09, .92)	.56 (.07, 1.04)	.36 (-.03, .75)
			Std. effect	.08	.24	.32	.21
			p-value	.58	.10	.02	.07
		Mother Hispanic	Effect (con. interval)	.02 (-.36, .40)	-.07 (-.47, .34)	-.14 (-.52, .25)	-.06 (-.36, .25)
			Std. effect	.01	-.04	-.08	-.03
			p-value	.92	.75	.49	.72
			N (deg. freedom)	929 (883)	921 (876)	920 (875)	2770 (972)
2	Non-food economic hardship index	High-cash gift	Effect (con. interval)	-.06 (-.30, .18)	.08 (-.16, .33)	-.10 (-.28, .09)	-.02 (-.19, .14)
			Std. effect	-.05	.07	-.11	-.03
			p-value	.61	.50	.30	.78
		High-cash X Mother Hispanic	Effect (con. interval)	.26 (-.07, .59)	.11 (-.23, .45)	.29 (.04, .55)	.22 (-.02, .46)
			Std. effect	.23	.10	.32	.22
			p-value	.12	.53	.02	.07

		Mother Hispanic	Effect (con. interval)	-.07 (-.32, .18)	.11 (-.15, .36)	-.13 (-.33, .07)	-.03 (-.21, .14)
			Std. effect	-.06	.10	-.14	-.04
			p-value	.58	.42	.20	.73
			N (deg. freedom)	930 (884)	921 (876)	922 (877)	2773 (972)
2	Expense worry	High-cash gift	Effect (con. interval)	.07 (-.26, .40)	.01 (-.32, .33)	-.06 (-.39, .26)	.01 (-.25, .26)
			Std. effect	.04	.00	-.04	.00
			p-value	.67	.97	.70	.95
		High-cash X Mother Hispanic	Effect (con. interval)	.16 (-.29, .62)	.30 (-.17, .76)	.31 (-.15, .76)	.26 (-.10, .61)
			Std. effect	.10	.18	.19	.16
			p-value	.48	.21	.19	.16
		Mother Hispanic	Effect (con. interval)	.10 (-.26, .46)	.09 (-.27, .46)	.08 (-.28, .44)	.09 (-.17, .36)
			Std. effect	.06	.06	.05	.06
			p-value	.58	.61	.67	.50
			N (deg. freedom)	930 (884)	919 (874)	919 (874)	2768 (972)
Panel 3: Maternal Psychological Distress							
3	Perceived stress index	High-cash gift	Effect (con. interval)	.50 (-.73, 1.73)	.41 (-.85, 1.67)	.87 (-.55, 2.29)	.61 (-.39, 1.60)
			Std. effect	.08	.07	.12	.09
			p-value	.42	.52	.23	.23
		High-cash X Mother Hispanic	Effect (con. interval)	.13 (-1.53, 1.79)	.44 (-1.22, 2.11)	.35 (-1.52, 2.21)	.30 (-1.03, 1.64)
			Std. effect	.02	.07	.05	.05
			p-value	.88	.60	.72	.66
		Mother Hispanic	Effect (con. interval)	1.11 (-.26, 2.47)	.58 (-.78, 1.94)	-.13 (-1.69, 1.42)	.53 (-.58, 1.64)
			Std. effect	.17	.09	-.02	.08
			p-value	.11	.40	.87	.35
			N (deg. freedom)	930 (884)	920 (875)	921 (876)	2771 (973)
3	Parenting stress index	High-cash gift	Effect (con. interval)	.65 (-.02, 1.31)	.54 (-.14, 1.22)		.59 (.03, 1.16)
			Std. effect	.18	.15		.17
			p-value	.06	.12		.04
		High-cash X Mother Hispanic	Effect (con. interval)	-.23 (-1.18, .72)	.11 (-.86, 1.09)		-.06 (-.88, .76)
			Std. effect	-.07	.03		-.02
			p-value	.64	.82		.89
		Mother Hispanic	Effect (con. interval)	2.18 (1.39, 2.97)	1.45 (.67, 2.23)		1.81 (1.13, 2.49)
			Std. effect	.62	.41		.51
			p-value	.00	.00		.00
			N (deg. freedom)	929 (883)	918 (873)		1847 (964)
3	Maternal depression (PHQ-8)	High-cash gift	Effect (con. interval)	.12 (-.72, .96)	.45 (-.37, 1.27)	-.09 (-.91, .73)	.18 (-.46, .81)
			Std. effect	.03	.12	-.02	.05
			p-value	.78	.28	.83	.58
		High-cash X Mother Hispanic	Effect (con. interval)	.65 (-.52, 1.82)	.16 (-.92, 1.24)	.62 (-.50, 1.73)	.46 (-.41, 1.33)
			Std. effect	.16	.04	.13	.11
			p-value	.28	.77	.28	.30

		Mother Hispanic	Effect (con. interval)	.54 (-.38, 1.46)	.52 (-.32, 1.36)	.42 (-.49, 1.34)	.51 (-.18, 1.20)
			Std. effect	.13	.13	.09	.12
			p-value	.25	.22	.37	.14
			N (deg. freedom)	930 (884)	919 (874)	919 (874)	2768 (973)
3	Maternal anxiety (GAD-7)	High-cash gift	Effect (con. interval)		.24 (-.57, 1.05)	.26 (-.52, 1.04)	.27 (-.39, .93)
			Std. effect		.06	.06	.07
			p-value		.56	.52	.43
		High-cash X Mother Hispanic	Effect (con. interval)		.46 (-.60, 1.52)	.31 (-.77, 1.40)	.37 (-.52, 1.26)
			Std. effect		.12	.07	.09
			p-value		.40	.57	.41
		Mother Hispanic	Effect (con. interval)		.01 (-.83, .86)	-.23 (-1.09, .63)	-.08 (-.78, .62)
			Std. effect		.00	-.05	-.02
			p-value		.97	.60	.83
			N (deg. freedom)		919 (874)	921 (876)	1840 (956)
3	Maternal anxiety (Beck Anxiety Inventory)	High-cash gift	Effect (con. interval)	1.84 (.32, 3.35)		.30 (-1.10, 1.70)	1.05 (-.20, 2.29)
			Std. effect	.28		.04	.15
			p-value	.02		.68	.10
		High-cash X Mother Hispanic	Effect (con. interval)	.01 (-2.06, 2.08)		-.03 (-2.05, 1.99)	.01 (-1.75, 1.76)
			Std. effect	.00		-.00	.00
			p-value	.99		.98	1.00
		Mother Hispanic	Effect (con. interval)	2.15 (.71, 3.59)		1.30 (-.38, 2.99)	1.74 (.41, 3.07)
			Std. effect	.33		.16	.24
			p-value	.00		.13	.01
			N (deg. freedom)	930 (884)		919 (874)	1849 (967)
3	Physiological stress (Ln hair cortisol)	High-cash gift	Effect (con. interval)	.11 (-.44, .66)			.11 (-.44, .66)
			Std. effect	.08			.08
			p-value	.69			.69
		High-cash X Mother Hispanic	Effect (con. interval)	-.12 (-.80, .55)			-.12 (-.80, .55)
			Std. effect	-.09			-.09
			p-value	.72			.72
		Mother Hispanic	Effect (con. interval)	-.66 (-1.12, -.20)			-.66 (-1.12, -.20)
			Std. effect	-.48			-.48
			p-value	.01			.01
			N (deg. freedom)	364 (318)			364 (363)
Panel 4: Interparental Relationship Quality							
4	Co-parenting relationship quality	High-cash gift	Effect (con. interval)	-.37 (-1.16, .42)	-.27 (-1.07, .54)		-.32 (-.99, .36)
			Std. effect	-.13	-.10		-.11
			p-value	.36	.52		.36
		High-cash X Mother Hispanic	Effect (con. interval)	-.33 (-1.29, .63)	-.39 (-1.34, .56)		-.35 (-1.17, .47)
			Std. effect	-.11	-.14		-.12
			p-value	.50	.42		.40

		Mother Hispanic	Effect (con. interval)	.57 (-.21, 1.36)	.48 (-.19, 1.16)		.53 (-.08, 1.15)
			Std. effect	.20	.18		.19
			p-value	.15	.16		.09
			N (deg. freedom)	720 (674)	663 (618)		1383 (802)
4	Romantic relationship quality index	High-cash gift	Effect (con. interval)	-.15 (-1.13, .84)	-.25 (-1.05, .55)	-.81 (-1.67, .06)	-.51 (-1.13, .11)
			Std. effect	-.04	-.09	-.22	-.15
			p-value	.77	.54	.07	.11
		High-cash X Mother Hispanic	Effect (con. interval)	-.98 (-2.28, .32)	-.07 (-1.10, .95)	.02 (-1.15, 1.18)	-.22 (-1.03, .59)
			Std. effect	-.28	-.03	.00	-.06
			p-value	.14	.89	.98	.59
		Mother Hispanic	Effect (con. interval)	.30 (-.72, 1.33)	-.34 (-1.07, .39)	-.33 (-1.17, .52)	-.15 (-.74, .44)
			Std. effect	.09	-.13	-.09	-.05
			p-value	.56	.36	.45	.62
			N (deg. freedom)	572 (526)	512 (467)	793 (748)	1877 (900)
4	Ever cut/bruised/seriously hurt by partner	High-cash gift	Effect (con. interval)	-.06 (-.14, .02)	-.00 (-.05, .05)		-.03 (-.09, .02)
			Std. effect	-.22	-.02		-.13
			p-value	.12	.93		.19
		High-cash X Mother Hispanic	Effect (con. interval)	.08 (-.02, .18)	-.00 (-.05, .05)		.04 (-.02, .10)
			Std. effect	.28	-.03		.14
			p-value	.12	.90		.19
		Mother Hispanic	Effect (con. interval)	-.08 (-.17, .01)	-.01 (-.05, .03)		-.05 (-.10, .00)
			Std. effect	-.30	-.09		-.22
			p-value	.07	.60		.05
			N (deg. freedom)	572 (526)	511 (466)		1083 (770)
4	Frequency of arguing	High-cash gift	Effect (con. interval)	-.14 (-.42, .14)	.20 (-.09, .49)		.02 (-.20, .23)
			Std. effect	-.14	.24		.04
			p-value	.32	.18		.87
		High-cash X Mother Hispanic	Effect (con. interval)	.20 (-.17, .57)	-.17 (-.53, .18)		.03 (-.23, .30)
			Std. effect	.20	-.21		.02
			p-value	.28	.33		.80
		Mother Hispanic	Effect (con. interval)	-.15 (-.46, .15)	.07 (-.20, .33)		-.07 (-.28, .13)
			Std. effect	-.15	.08		-.07
			p-value	.33	.62		.49
			N (deg. freedom)	566 (520)	512 (467)		1078 (766)
Panel 5: Parenting Quality							
5	Parent-child activities index	High-cash gift	Effect (con. interval)	.85 (.32, 1.38)	.78 (.19, 1.36)	.56 (.03, 1.08)	.73 (.29, 1.17)
			Std. effect	.32	.26	.22	.27
			p-value	.00	.01	.04	.00
		High-cash X Mother Hispanic	Effect (con. interval)	-.84 (-1.58, -.10)	-.57 (-1.39, .25)	-.33 (-1.02, .36)	-.58 (-1.18, .01)
			Std. effect	-.31	-.19	-.13	-.21
			p-value	.03	.17	.35	.06

		Mother Hispanic	Effect (con. interval)	-.43 (-1.03, .17)	-.57 (-1.20, .06)	-.41 (-.95, .14)	-.47 (-.93, -.00)
			Std. effect	-.16	-.19	-.16	-.17
			p-value	.16	.08	.14	.05
			N (deg. freedom)	929 (883)	919 (874)	915 (870)	2763 (971)
5	Parent-child interaction (PICCOLO)	High-cash gift	Effect (con. interval)	.43 (-.86, 1.73)			.43 (-.86, 1.73)
			Std. effect	.08			.08
			p-value	.51			.51
		High-cash X Mother Hispanic	Effect (con. interval)	.82 (-1.13, 2.76)			.82 (-1.13, 2.76)
			Std. effect	.15			.15
			p-value	.41			.41
		Mother Hispanic	Effect (con. interval)	-2.38 (-4.13, -.63)			-2.38 (-4.13, -.63)
			Std. effect	-.43			-.43
			p-value	.01			.01
			N (deg. freedom)	543 (497)			543 (542)
5	Spanking discipline strategy	High-cash gift	Effect (con. interval)	.04 (-.03, .11)	-.01 (-.10, .07)	-.04 (-.13, .05)	-.01 (-.07, .05)
			Std. effect	.17	-.03	-.10	-.01
			p-value	.26	.76	.38	.69
		High-cash X Mother Hispanic	Effect (con. interval)	-.05 (-.13, .03)	-.05 (-.15, .05)	.00 (-.10, .11)	-.03 (-.10, .04)
			Std. effect	-.22	-.13	.01	-.10
			p-value	.19	.31	.95	.44
		Mother Hispanic	Effect (con. interval)	-.05 (-.11, .02)	-.02 (-.11, .07)	-.09 (-.18, .00)	-.05 (-.11, .01)
			Std. effect	-.20	-.05	-.22	-.15
			p-value	.15	.68	.05	.09
			N (deg. freedom)	596 (463)	914 (780)	917 (782)	2427 (959)

Notes: Data collection occurred in July 2019 to June 2020 for age 1, July 2020 to July 2021 for age 2, and July 2021 to July 2022 for age 3. Each block of rows presents for each outcome three coefficients from the same regression model: 1) high-cash gift group assignment (treatment status); 2) an interaction term between high-gift group assignment and mother self-identifying as Hispanic (of any race) at the time of random assignment, and 3 mother self-identifying as Hispanic (of any race) at the time of random assignment. The reference group in this analysis is mothers who self-identified as Black non-Hispanic at baseline. Some mothers identified as White (10%) or another non-Hispanic racial category (9%) at baseline. Given the small sample sizes, the interaction of these groups with the high-cash gift group were included in the regression but excluded from the results reported in this table. As a result, the results compare treatment impacts for Black non-Hispanic mothers to Hispanic mothers of any race. For all coefficients the raw coefficient in provided with confidence intervals in parentheses; the standardized coefficient effect size; and the *p*-value. The last row of each outcome presents number of observations and degrees of freedom. Covariates in all models include site fixed-effects, baseline covariates, child age at interview, and phone interview status. The "Pooled Sample" column presents estimates from analyses that pool observations across ages, adjust for age indicators, and cluster the standard error at the individual level. We report the degrees of freedom which is computed as the sample size minus the number of parameters estimated in the model. This statistic is complicated in the pooled sample because we cluster the standard error to adjust for non-independence. For simplicity, we report the default degrees of freedom reported in most software, which is the number of clusters minus one. The *p*-value comes from two-sided tests that do not correct for multiple outcomes. Household incomes across all years are inflation-adjusted to 2019 dollars, and the poverty line is based on the 2019 U.S. Census poverty threshold. Income-to-needs is the household income divided by the poverty line for a given family size and composition. Income and income-to-needs have been truncated at the 99th percentile. PHQ-8=Personal Health Questionnaire Depression scale. GAD-7=General Anxiety Disorder-7. PICCOLO=Parenting Interaction with Children: Checklist of Observations Linked to Outcomes.

Appendix Table 15 Summary of ITT estimates of impacts of the BFY high-cash gift on family well-being and family processes measures moderated by reported high household income, measured as an income greater than sample median

Family	Outcome	Var.		Age 1	Age 2	Age 3	Pooled Sample
Panel 1: Economic Resources							
1	Income-to-needs ratio (including the BFY gifts)	High-cash gift	Effect (con. interval)			.08 (-.05, .20)	.16 (.02, .30)
			Std. effect			.09	.19
			p-value			.25	.02
		High-cash X Higher Income	Effect (con. interval)			.06 (-.14, .27)	-.06 (-.28, .17)
			Std. effect			.08	-.07
			p-value			.54	.62
		Higher Income	Effect (con. interval)			.26 (.13, .40)	.35 (.20, .50)
			Std. effect			.31	.41
			p-value			.00	.00
			N (deg. freedom)			922 (877)	922 (877)
1	Household income with gift ($1000s, in 2019 dollars)	High-cash gift	Effect (con. interval)			1.98 (-1.71, 5.67)	4.00 (.22, 7.77)
			Std. effect			.08	.16
			p-value			.29	.04
		High-cash X Higher Income	Effect (con. interval)			2.14 (-3.99, 8.27)	-1.19 (-7.87, 5.48)
			Std. effect			.09	-.05
			p-value			.49	.73
		Higher Income	Effect (con. interval)			8.33 (4.22, 12.44)	10.83 (6.48, 15.18)
			Std. effect			.33	.42
			p-value			.00	.00
			N (deg. freedom)			922 (877)	922 (877)
Panel 2: Economic Pressure							
2	Food insecurity index	High-cash gift	Effect (con. interval)	.21 (-.13, .54)	.04 (-.30, .38)	-.03 (-.34, .29)	.08 (-.17, .34)
			Std. effect	.12	.02	-.02	.05
			p-value	.22	.81	.87	.53
		High-cash X Higher Income	Effect (con. interval)	.03 (-.43, .49)	-.01 (-.48, .45)	.16 (-.30, .61)	.06 (-.30, .42)
			Std. effect	.02	-.01	.09	.04
			p-value	.90	.96	.50	.73
		Higher Income	Effect (con. interval)	-.22 (-.52, .08)	-.15 (-.45, .15)	-.13 (-.43, .17)	-.17 (-.40, .07)
			Std. effect	-.13	-.09	-.07	-.10
			p-value	.15	.32	.40	.16
			N (deg. freedom)	929 (883)	921 (876)	920 (875)	2770 (972)
2	Non-food economic hardship index	High-cash gift	Effect (con. interval)	.11 (-.11, .32)	.02 (-.21, .25)	.04 (-.12, .21)	.06 (-.09, .22)
			Std. effect	.09	.02	.05	.06
			p-value	.33	.85	.59	.43
		High-cash X Higher Income	Effect (con. interval)	-.12 (-.43, .19)	.10 (-.22, .43)	-.03 (-.27, .21)	-.02 (-.24, .20)
			Std. effect	-.10	.09	-.04	-.02
			p-value	.46	.53	.78	.86

		Higher Income	Effect (con. interval)	.07 (-.13, .28)	.09 (-.11, .29)	.05 (-.11, .21)	.07 (-.07, .21)
			Std. effect	.07	.08	.05	.07
			p-value	.47	.40	.57	.30
			N (deg. freedom)	930 (884)	921 (876)	922 (877)	2773 (972)
2	Expense worry	High-cash gift	Effect (con. interval)	.08 (-.23, .39)	.01 (-.30, .32)	-.13 (-.43, .17)	-.01 (-.25, .22)
			Std. effect	.05	.00	-.08	-.01
			p-value	.60	.96	.39	.91
		High-cash X Higher Income	Effect (con. interval)	.22 (-.21, .64)	.26 (-.18, .69)	.48 (.05, .90)	.32 (-.01, .65)
			Std. effect	.13	.16	.29	.20
			p-value	.33	.25	.03	.06
		Higher Income	Effect (con. interval)	-.13 (-.43, .16)	-.03 (-.31, .26)	-.26 (-.55, .02)	-.14 (-.36, .07)
			Std. effect	-.08	-.02	-.16	-.09
			p-value	.36	.86	.07	.20
			N (deg. freedom)	930 (884)	919 (874)	919 (874)	2768 (972)
Panel 3: Maternal Psychological Distress							
3	Perceived stress index	High-cash gift	Effect (con. interval)	.25 (-.89, 1.39)	.37 (-.78, 1.52)	.61 (-.66, 1.89)	.42 (-.50, 1.33)
			Std. effect	.04	.06	.08	.06
			p-value	.67	.53	.34	.37
		High-cash X Higher Income	Effect (con. interval)	.78 (-.83, 2.38)	.34 (-1.27, 1.95)	.26 (-1.57, 2.10)	.48 (-.82, 1.79)
			Std. effect	.12	.05	.04	.07
			p-value	.34	.68	.78	.47
		Higher Income	Effect (con. interval)	-.40 (-1.48, .69)	.08 (-1.01, 1.16)	-.05 (-1.34, 1.24)	-.14 (-1.04, .76)
			Std. effect	-.06	.01	-.01	-.02
			p-value	.48	.89	.94	.76
			N (deg. freedom)	930 (884)	920 (875)	921 (876)	2771 (973)
3	Parenting stress index	High-cash gift	Effect (con. interval)	.46 (-.22, 1.14)	.57 (-.12, 1.26)		.51 (-.08, 1.09)
			Std. effect	.13	.16		.14
			p-value	.19	.11		.09
		High-cash X Higher Income	Effect (con. interval)	.26 (-.65, 1.17)	-.07 (-1.02, .88)		.10 (-.69, .90)
			Std. effect	.07	-.02		.03
			p-value	.58	.88		.80
		Higher Income	Effect (con. interval)	-.25 (-.87, .38)	-.11 (-.75, .53)		-.18 (-.73, .36)
			Std. effect	-.07	-.03		-.05
			p-value	.44	.74		.51
			N (deg. freedom)	929 (883)	918 (873)		1847 (964)
3	Maternal depression (PHQ-8)	High-cash gift	Effect (con. interval)	-.10 (-.90, .70)	.35 (-.45, 1.15)	-.26 (-1.01, .48)	-.00 (-.60, .59)
			Std. effect	-.02	.09	-.06	.00
			p-value	.81	.39	.49	1.00
		High-cash X Higher Income	Effect (con. interval)	.60 (-.49, 1.70)	.07 (-1.01, 1.15)	.40 (-.68, 1.49)	.36 (-.48, 1.20)
			Std. effect	.15	.02	.09	.08
			p-value	.28	.89	.47	.40

		Higher Income	Effect (con. interval)	-.51 (-1.22, .21)	.15 (-.48, .79)	.01 (-.74, .76)	-.12 (-.65, .41)
			Std. effect	-.12	.04	.00	-.03
			p-value	.16	.64	.98	.66
			N (deg. freedom)	930 (884)	919 (874)	919 (874)	2768 (973)
3	Maternal anxiety (GAD-7)	High-cash gift	Effect (con. interval)		.28 (-.49, 1.06)	.07 (-.64, .79)	.19 (-.43, .81)
			Std. effect		.07	.02	.05
			p-value		.47	.84	.55
		High-cash X Higher Income	Effect (con. interval)		.10 (-.97, 1.18)	.19 (-.86, 1.24)	.13 (-.75, 1.02)
			Std. effect		.03	.04	.03
			p-value		.85	.73	.77
		Higher Income	Effect (con. interval)		.08 (-.54, .70)	.47 (-.24, 1.19)	.29 (-.27, .85)
			Std. effect		.02	.11	.07
			p-value		.79	.19	.31
			N (deg. freedom)		919 (874)	921 (876)	1840 (956)
3	Maternal anxiety (Beck Anxiety Inventory)	High-cash gift	Effect (con. interval)	1.95 (.46, 3.44)		-.08 (-1.40, 1.25)	.90 (-.29, 2.10)
			Std. effect	.30		-.01	.14
			p-value	.01		.91	.14
		High-cash X Higher Income	Effect (con. interval)	-.35 (-2.34, 1.64)		.59 (-1.34, 2.52)	.14 (-1.50, 1.79)
			Std. effect	-.05		.07	.01
			p-value	.73		.55	.87
		Higher Income	Effect (con. interval)	-.73 (-1.79, .33)		.17 (-1.12, 1.46)	-.29 (-1.27, .68)
			Std. effect	-.11		.02	-.05
			p-value	.18		.80	.55
			N (deg. freedom)	930 (884)		919 (874)	1849 (967)
3	Physiological stress (ln hair cortisol)	High-cash gift	Effect (con. interval)	-.27 (-.68, .14)			-.27 (-.68, .14)
			Std. effect	-.20			-.20
			p-value	.20			.20
		High-cash X Higher Income	Effect (con. interval)	.64 (.03, 1.25)			.64 (.03, 1.25)
			Std. effect	.47			.47
			p-value	.04			.04
		Higher Income	Effect (con. interval)	-.42 (-.82, -.02)			-.42 (-.82, -.02)
			Std. effect	-.31			-.31
			p-value	.04			.04
			N (deg. freedom)	364 (318)			364 (363)
Panel 4: Interparental Relationship Quality							
4	Co-parenting relationship quality	High-cash gift	Effect (con. interval)	-.39 (-1.13, .35)	-.26 (-.94, .42)		-.31 (-.92, .29)
			Std. effect	-.13	-.09		-.11
			p-value	.30	.45		.31
		High-cash X Higher Income	Effect (con. interval)	-.05 (-1.01, .90)	.02 (-.89, .94)		-.01 (-.81, .79)
			Std. effect	-.02	.01		-.00
			p-value	.91	.96		.98

		Higher Income	Effect (con. interval)	.08 (-.49, .65)	.12 (-.45, .68)		.10 (-.37, .57)
			Std. effect	.03	.04		.04
			p-value	.79	.69		.67
			N (deg. freedom)	720 (674)	663 (618)		1383 (802)
4	Romantic relationship quality index	High-cash gift	Effect (con. interval)	-.56 (-1.46, .34)	-.14 (-.99, .72)	-.62 (-1.44, .19)	-.51 (-1.11, .09)
			Std. effect	-.16	-.05	-.17	-.15
			p-value	.22	.75	.13	.09
		High-cash X Higher Income	Effect (con. interval)	.51 (-.81, 1.83)	-.41 (-1.47, .64)	.16 (-.99, 1.31)	.11 (-.69, .92)
			Std. effect	.14	-.15	.04	.02
			p-value	.45	.44	.78	.78
		Higher Income	Effect (con. interval)	-.18 (-1.01, .64)	.50 (-.14, 1.15)	.38 (-.35, 1.11)	.23 (-.28, .75)
			Std. effect	-.05	.19	.10	.08
			p-value	.67	.13	.31	.38
			N (deg. freedom)	572 (526)	512 (468)	793 (748)	1877 (900)
4	Ever cut/bruised/seriously hurt by partner	High-cash gift	Effect (con. interval)	-.02 (-.09, .05)	.02 (-.02, .06)		-.00 (-.05, .04)
			Std. effect	-.07	.18		.04
			p-value	.57	.35		.93
		High-cash X Higher Income	Effect (con. interval)	.01 (-.10, .11)	-.01 (-.07, .04)		.00 (-.06, .06)
			Std. effect	.03	-.11		-.02
			p-value	.88	.65		.96
		Higher Income	Effect (con. interval)	-.03 (-.10, .04)	-.00 (-.04, .03)		-.02 (-.06, .02)
			Std. effect	-.11	-.03		-.06
			p-value	.39	.86		.43
			N (deg. freedom)	572 (526)	511 (467)		1083 (770)
4	Frequency of arguing	High-cash gift	Effect (con. interval)	-.10 (-.35, .15)	.02 (-.22, .26)		-.03 (-.22, .15)
			Std. effect	-.10	.03		-.03
			p-value	.43	.85		.71
		High-cash X Higher Income	Effect (con. interval)	.14 (-.21, .49)	.18 (-.12, .49)		.17 (-.08, .42)
			Std. effect	.14	.22		.19
			p-value	.44	.24		.18
		Higher Income	Effect (con. interval)	-.03 (-.28, .21)	-.05 (-.26, .17)		-.05 (-.21, .12)
			Std. effect	-.03	-.06		-.05
			p-value	.78	.67		.59
			N (deg. freedom)	566 (520)	512 (468)		1078 (766)
Panel 5: Parenting Quality							
5	Parent-child activities index	High-cash gift	Effect (con. interval)	.58 (.08, 1.08)	.46 (-.11, 1.03)	.51 (.04, .98)	.51 (.10, .92)
			Std. effect	.22	.15	.20	.19
			p-value	.02	.11	.03	.01
		High-cash X Higher Income	Effect (con. interval)	-.22 (-.93, .48)	-.07 (-.86, .71)	-.13 (-.82, .55)	-.13 (-.72, .45)
			Std. effect	-.08	-.02	-.05	-.05
			p-value	.54	.86	.70	.65

		Higher Income	Effect (con. interval)	.28 (-.22, .77)	.46 (-.10, 1.01)	-.00 (-.47, .47)	.25 (-.17, .66)
			Std. effect	.10	.15	-.00	.09
			p-value	.27	.11	1.00	.24
			N (deg. freedom)	929 (883)	919 (874)	915 (870)	2763 (971)
5	Parent-child interaction (PICCOLO)	High-cash gift	Effect (con. interval)	.75 (-.53, 2.03)			.75 (-.53, 2.03)
			Std. effect	.14			.14
			p-value	.25			.25
		High-cash X Higher Income	Effect (con. interval)	-.87 (-2.83, 1.10)			-.87 (-2.83, 1.10)
			Std. effect	-.16			-.16
			p-value	.39			.39
		Higher Income	Effect (con. interval)	.50 (-.80, 1.79)			.50 (-.80, 1.79)
			Std. effect	.09			.09
			p-value	.45			.45
			N (deg. freedom)	543 (497)			543 (542)
5	Spanking discipline strategy	High-cash gift	Effect (con. interval)	.01 (-.04, .07)	-.01 (-.08, .06)	-.01 (-.08, .07)	-.00 (-.05, .05)
			Std. effect	.06	-.03	-.02	-.00
			p-value	.63	.75	.84	.89
		High-cash X Higher Income	Effect (con. interval)	.00 (-.08, .09)	-.09 (-.19, .01)	-.04 (-.14, .07)	-.05 (-.12, .03)
			Std. effect	.01	-.22	-.09	-.11
			p-value	.98	.08	.50	.21
		Higher Income	Effect (con. interval)	.03 (-.02, .09)	.06 (-.01, .13)	.02 (-.05, .09)	.04 (-.01, .09)
			Std. effect	.14	.16	.06	.12
			p-value	.23	.08	.50	.09
			N (deg. freedom)	596 (550)	914 (869)	917 (872)	2427 (959)

Notes: Data collection occurred in July 2019 to June 2020 for age 1, July 2020 to July 2021 for age 2, and July 2021 to July 2022 for age 3. Each block of rows presents for each outcome three coefficients from the same regression model: 1) high-cash gift group assignment (treatment status); 2) an interaction term between high-gift group assignment and the mother reporting an income that was in the top 50% of the baseline sample income distribution at the time of random assignment, and 3) the mother reporting an income that was in the top 50% of the baseline sample income distribution. The reference group in this analysis is mothers who reported a household income in the lower half of the income distribution at baseline. A small number of mothers reported not knowing their household income during the baseline survey. Given the small sample size of this group, the interaction of this group with the high-cash gift group was included in the regression but excluded from the results reported in this table. For all coefficients the raw coefficient is provided with confidence intervals in parentheses; the standardized coefficient effect size; and the *p*-value. The last row of each outcome presents number of observations and degrees of freedom. Covariates in all models include site fixed-effects, baseline covariates, child age at interview, and phone interview status. The "Pooled Sample" column presents estimates from analyses that pool observations across ages, adjust for age indicators, and cluster the standard error at the individual level. We report the degrees of freedom which is computed as the sample size minus the number of parameters estimated in the model. This statistic is complicated in the pooled sample because we cluster the standard error to adjust for non-independence. For simplicity, we report the default degrees of freedom reported in most software, which is the number of clusters minus one. The *p*-value comes from two-sided tests that do not correct for multiple outcomes. Household incomes across all years are inflation-adjusted to 2019 dollars, and the poverty line is based on the 2019 U.S. Census poverty threshold. Income-to-needs is the household income divided by the poverty line for a given family size and composition. Income and income-to-needs have been truncated at the 99th percentile. PHQ-8=Personal Health Questionnaire Depression scale. GAD-7=General Anxiety Disorder-7. PICCOLO=Parenting Interaction with Children: Checklist of Observations Linked to Outcomes.

Supplementary References

1. Gennetian, L. A. et al. Effects of a monthly unconditional cash transfer starting at birth on family investments among US families with low income. *Nat. Hum. Behav.* **8,** 1514–1529 (2024).
2. U.S. Department of Agriculture. U.S. Household Food Security Survey Module: Six Item Short Form. Retrieved August 5, 2024, from https://www.ers.usda.gov/media/8282/short2012.pdf (2012).
3. Kling, J. R., Liebman, J. B. & Katz, L. F. Experimental analysis of neighborhood effects. *Econometrica*, **75,** 83–119 (2007).
4. Cohen, S., Kamarck, T. & Mermelstein, R. A global measure of perceived stress. *J. Health Soc. Behav.* **24,** 385–396 (1983).
5. Cohen, S., & Williamson, G. M. Perceived stress in a probability sample of the United States in *The Social Psychology of Health: Claremont Symposium on Applied Social Psychology* (eds. Spacapan, S., & Oskamp, S.) pp. 31– 67 (Sage, Newbury Park, CA, 1988).
6. Hewitt, P. L., Flett, G. L. & Mosher, S. W. The Perceived Stress Scale: factor structure and relation to depression symptoms in a psychiatric sample. *J. Psychopathol. Behav. Assess.* **14,** 247–257 (1992).
7. Schickedanz, A., Halfon, N., Sastry, N. & Chung, P. J. Parents' adverse childhood experiences and their children's behavioral health problems. *Pediatrics* **142,** e20180023 (2018).
8. Kroenke, K. et al. The PHQ-8 as a measure of current depression in the general population. *J. Aff. Disord.* **114,** 163–173 (2009).
9. Spitzer, R. L., Kroenke, K., Williams, J. B. & Löwe, B. A brief measure for assessing generalized anxiety disorder: the GAD-7. *Arch. Intern. Med.* **166,** 1092–1097 (2006).
10. Beck, A. T., Epstein, N., Brown, G. & Steer, R. A. An inventory for measuring clinical anxiety: psychometric properties. *J. Consulting Clin. Psychol.*, **56,** 893 (1988).
11. Creamer, M., Foran, J. & Bell, R. The Beck Anxiety Inventory in a non-clinical sample. *Behav. Res. Therapy* **33,** 477–485 (1995).
12. Meyer, J., Novak, M., Hamel, A. & Rosenberg, K. Extraction and analysis of cortisol from human and monkey hair. *J. Vis. Exp.* **83,** e50882 (2014).
13. McLanahan, S. & Beck, A. N. Parental relationships in fragile families. *Future Child.* **20,** 17–37 (2010).
14. Turney, K. Hopelessly devoted? relationship quality during and after incarceration. *J. Marriage Fam.* **77,** 480–495 (2015).
15. Rodriguez, E. T. & Tamis-LeMonda, C. S. Trajectories of the home learning environment across the first 5 years: associations with children's vocabulary and literacy skills at prekindergarten. *Child Dev.* **82,** 1058–1075 (2011).
16. Roggman, L. A., Cook, G. A., Innocenti, M. S., Jump Norman, V. & Christiansen, K. Parenting interactions with children: checklist of observations linked to outcomes (PICCOLO) in diverse ethnic groups. *Infant Ment. Health J.* **34,** 290–306 (2013).
17. Reichman, N. E., Teitler, J. O., Garfinkel, I. & McLanahan, S. S. Fragile Families: sample and design. *Child. Youth Serv. Rev.* **23,** 303–326 (2001).
18. Westfall, P. H. & Young, S. S. *Resampling-Based Multiple Testing: Examples and Methods for p-Value Adjustment* (Wiley, New York, 1993).
19. Masarik, A. S. & Conger, R. D. Stress and child development: a review of the Family Stress Model. *Curr. Opin. Psychol.* **13,** 85–90 (2017).
