# Supplementary material for: Effects of unconditional cash transfers on family processes and wellbeing among mothers with low incomes
Source: Nat Commun. 2025 Aug 13;16:7517. doi: 10.1038/s41467-025-62438-x (PMC12350725; doi:10.1038/s41467-025-62438-x)
Supplement: Supplementary file 2 — Reporting Summary [file 41467_2025_62438_MOESM2_ESM.pdf]

nature portfolio

Corresponding author(s): Katherine Magnuson

Last updated by author(s): 4/8/2025

Reporting Summary

Nature Portfolio wishes to improve the reproducibility of the work that we publish. This form provides structure for consistency and transparency in reporting. For further information on Nature Portfolio policies, see our Editorial Policies and the Editorial Policy Checklist.

Statistics

For all statistical analyses, confirm that the following items are present in the figure legend, table legend, main text, or Methods section.

n/a	Confirmed	
☐	☒	The exact sample size (*n*) for each experimental group/condition, given as a discrete number and unit of measurement
☐	☒	A statement on whether measurements were taken from distinct samples or whether the same sample was measured repeatedly
☐	☒	The statistical test(s) used AND whether they are one- or two-sided<br>*Only common tests should be described solely by name; describe more complex techniques in the Methods section.*
☐	☒	A description of all covariates tested
☐	☒	A description of any assumptions or corrections, such as tests of normality and adjustment for multiple comparisons
☐	☒	A full description of the statistical parameters including central tendency (e.g. means) or other basic estimates (e.g. regression coefficient) AND variation (e.g. standard deviation) or associated estimates of uncertainty (e.g. confidence intervals)
☐	☒	For null hypothesis testing, the test statistic (e.g. *F*, *t*, *r*) with confidence intervals, effect sizes, degrees of freedom and *P* value noted<br>*Give P values as exact values whenever suitable.*
☒	☐	For Bayesian analysis, information on the choice of priors and Markov chain Monte Carlo settings
☒	☐	For hierarchical and complex designs, identification of the appropriate level for tests and full reporting of outcomes
☐	☒	Estimates of effect sizes (e.g. Cohen's *d*, Pearson's *r*), indicating how they were calculated

Our web collection on statistics for biologists contains articles on many of the points above.

Software and code

Policy information about availability of computer code

Data collection	Survey data collected by enumerators from the University of Michigan Survey Research Center, electronically, using Blaise v4.8 software.
Data analysis	Analyses of estimates and descriptive characteristics conducted using STATA V.18 statistical software as prepared by study team researchers. All stata code files used in this paper are available at: https://www.openicpsr.org/openicpsr/project/159422/version/V5/view?path=/openicpsr/159422/fcr:versions/V5/Magnuson-et-al-NComm-2025&type=folder. It can also be accessed through the BFY project code website: https://www.openicpsr.org/openicpsr/project/159422/version/V5/view

For manuscripts utilizing custom algorithms or software that are central to the research but not yet described in published literature, software must be made available to editors and reviewers. We strongly encourage code deposition in a community repository (e.g. GitHub). See the Nature Portfolio guidelines for submitting code & software for further information.

Data

Policy information about availability of data

All manuscripts must include a data availability statement. This statement should provide the following information, where applicable:

- Accession codes, unique identifiers, or web links for publicly available datasets
- A description of any restrictions on data availability
- For clinical datasets or third party data, please ensure that the statement adheres to our policy

Data are available as public use data at https://www.icpsr.umich.edu/web/DSDR/studies/37871. There is one covariate related to child age that is used in analyses but not publicly available due to HIPPA rules. This is noted in all analyses code files and results are not sensitive to it's exclusion.

Research involving human participants, their data, or biological material

Policy information about studies with human participants or human data. See also policy information about sex, gender (identity/presentation), and sexual orientation and race, ethnicity and racism.

Reporting on sex and gender	Birth parents were recruited in the study, and we therefore presume were assigned female at birth. We do not ask for participants' current gender identity, although we refer to the participants as mothers throughout.
Reporting on race, ethnicity, or other socially relevant groupings	The racial and ethnic diversity of the sample reflects that of the communities of the hospitals where mothers gave birth. The study sample is racially and ethnically diverse: per mothers' self-reports, 39% identify as Black, 42% Latina, <1% Asian/Pacific Islander, 1.5% Native American, 11% white, non-Hispanic, and 7% multiple races/other. To be included in the study all mothers had to report having a household income below the federal poverty threshold.
Population characteristics	Participating mothers and infants in the Baby's First Years study were recruited from 12 hospitals in four metropolitan areas: New York City, New Orleans, the greater Omaha metropolitan area, and the Twin Cities (Minneapolis and St. Paul). Selection of these metropolitan areas was guided by an aim to enroll a racially and ethnically diverse sample of low-income mothers across geographic regions that vary in cost of living and generosity of public services and safety-net programs. The racial and ethnic diversity of the sample reflects that of the communities of the hospitals where mothers gave birth. The mothers' average age at the start of the study was 27 years, and they had completely slightly less than 12 years of education. The study sample is racially and ethnically diverse: per mothers' self-reports, 39% identify as Black, 42% Latina, <1% Asian/Pacific Islander, 1.5% Native American, 11% white, non-Hispanic, and 7% multiple races/other. Approximately one out of three infants were first- time births for the mother, and one out of five mothers reported being married. Nearly 60% of mothers worked for pay while pregnant, and 92% reported plans to return to work. Forty-one percent reported that the biological father of the infant resided in the household. The average household income, at $22,313, is just above the federal poverty line for a family of three ($21,330 in 2019). Less than 13% of mothers reported receiving government cash assistance from the TANF program, whereas over 95% reported receiving some type of government benefit (SNAP, WIC [Special Supplemental Nutrition Program for Women, Infants, and Children], Head Start, other free childcare, Medicaid, housing assistance, unemployment benefits, or other). Eighty percent of families were net worth poor (defined as net worth less than one-fourth of the federal poverty line or having assets sufficient to meet basic needs for three months, as defined by the poverty line).
Recruitment	Study participants were recruited in hospital maternity wards shortly after giving birth. Participants were first approached about their interest in participating in a child development study. They were then asked about their interest in receiving a cash gift. Between May 2018 and June 2019, all 1,000 mother-infant dyads were recruited. Eligibility criteria for the study included (1) mother 18 years or older with the exception of Nebraska, where the age of consent was 19 years or older; (2) self-reported household income below the federal poverty threshold in the calendar year prior to the interview, counting the newborn; (3) healthy full-term birth (i.e., 37 weeks' gestation or greater; not in the NICU; no known developmental or neurological problems); (4) scheduled to be discharged into the custody of the birth mother; (5) living in the state of recruitment and not being highly likely to move to a different state or country in the next 12 months; and (6) proficiency in English or Spanish for the purposes of available child outcome measurement.
Ethics oversight	The institutional review boards of Teachers College (Protocol 18-210) and the New York State Psychiatric Institute (Protocol 7606) approved the study. It was registered at clinicaltrials.gov (NCT03593356; first posted July 2018, https://classic.clinicaltrials.gov/ct2/show/NCT03593356) and on the American Economic Association's registry (AEARCTR-0003262, first posted June 2019, https://www.socialscienceregistry.org/trials/3262). Informed consent was secured at the time of the study focal child's birth to participate in a longitudinal research study and for several components of data collection. Subsequently, consent was also provided at each wave of data collection.

Note that full information on the approval of the study protocol must also be provided in the manuscript.

Field-specific reporting

Please select the one below that is the best fit for your research. If you are not sure, read the appropriate sections before making your selection.

☐ Life sciences ☒ Behavioural & social sciences ☐ Ecological, evolutionary & environmental sciences

For a reference copy of the document with all sections, see nature.com/documents/nr-reporting-summary-flat.pdf

Behavioural & social sciences study design

All studies must disclose on these points even when the disclosure is negative.

Study description	This study examines the causal impact of a poverty reduction intervention via a randomized control trial of unconditional monthly cash gifts disbursed to low-income families with young children starting at the birth of the child. This study uses quantitative data from surveys and biological samples of hair to measure cortisol.
Research sample	The research study sample is n=1,000 mother-infant dyads recruited from maternity wards in 12 hospitals across four urban areas (New York City, New Orleans, Twin Cities, MN, and the greater Omaha area). Selection of these metropolitan areas was guided by an aim to enroll a racially and ethnically diverse sample of low-income mothers across geographic regions that vary in cost of living and generosity of public services and safety-net programs. The racial and ethnic diversity of the sample reflects that of the communities of the hospitals where mothers gave birth but is not formally representative of the communities or nationally representative.
Sampling strategy	Mother-infant dyads were recruited over the course of 12 months from approximately May 2018 through June 2019 in each of the four sites, distributed as approximately 290 in New Orleans, New York City and Omaha, and 150 in the Twin Cities. With an enrolled sample of n=1,000 mother-infant-dyads, and accounting for a predicted 20% attrition over longer-term follow-ups, the anticipated sample size of 800 dyads during subsequent waves of data collection was estimated to provide 80% power to detect a .207 standard deviation impact at p<.05 in a two-tailed test.
Data collection	Data for this study are drawn from a 30-minute baseline survey with consenting participants at study entry, and from an approximate 60-minute survey conducted at the three follow-up waves (approximately 1,2, and 3 years after the birth of the infant). The one-year follow-up survey was administered in person until March 2020 when COVID19 stopped in-person visits, and one-year data collection after March of 2020 was collected via a phone survey. Survey data enumerators were not blinded to study participants treatment status at birth at time of randomization of cash gift receipt and activating the debit card. However, survey data enumerators were not informed or reminded of treatment status thereafter.
Timing	The baseline survey co-occurred with study participant recruitment from approximately May 2018 to June 2019, with recruitment occurring continuously for 12 months. The wave 1 follow- up survey was conducted from July 2019 to July 2020 (children were 1-years old). The wave 2 follow-up survey was conducted from July 2020 to July 2021 (children were 2-years old). The wave 3 follow-up was conducted July 2021 to July 2022 (children were 3-years old).
Data exclusions	No data were excluded from participants who responded to the wave 1, 2, and/or wave 3 survey.
Non-participation	Study participant completion rates for the survey data were: 93%, 92%, and 92%, respectively, for follow-up waves at ages 1, 2 and 3 of the recruited infants.
Randomization	Upon receiving information about the opportunity to receive the unconditional monthly cash gift, study participants were randomized within site to a high-cash gift treatment (40% of the sample, n=400, receiving $333/month) or a low-cash gift treatment (60% of the study sample, n=600 receiving $20/month). The randomization was conducted through a prepopulated roster that assigned each sample identification number with a group assignment. This approach was used because it ensured the desired split of the cash gifts and could not be influenced by the interview staff. The baseline sample sizes were determined by an a priori power analysis with the goal of detecting an .20 effect size after several years (assuming 80% retention). This effect size was determined based on prior research in the field.

Reporting for specific materials, systems and methods

We require information from authors about some types of materials, experimental systems and methods used in many studies. Here, indicate whether each material, system or method listed is relevant to your study. If you are not sure if a list item applies to your research, read the appropriate section before selecting a response.

Materials & experimental systems

n/a	Involved in the study	
☒	☐	Antibodies
☒	☐	Eukaryotic cell lines
☒	☐	Palaeontology and archaeology
☒	☐	Animals and other organisms
☒	☐	Clinical data
☒	☐	Dual use research of concern
☒	☐	Plants

Methods

n/a	Involved in the study	
☒	☐	ChIP-seq
☒	☐	Flow cytometry
☒	☐	MRI-based neuroimaging

Plants

Seed stocks	*Report on the source of all seed stocks or other plant material used. If applicable, state the seed stock centre and catalogue number. If plant specimens were collected from the field, describe the collection location, date and sampling procedures.*
Novel plant genotypes	*Describe the methods by which all novel plant genotypes were produced. This includes those generated by transgenic approaches, gene editing, chemical/radiation-based mutagenesis and hybridization. For transgenic lines, describe the transformation method, the number of independent lines analyzed and the generation upon which experiments were performed. For gene-edited lines, describe the editor used, the endogenous sequence targeted for editing, the targeting guide RNA sequence (if applicable) and how the editor was applied.*
Authentication	*Describe any authentication procedures for each seed stock used or novel genotype generated. Describe any experiments used to assess the effect of a mutation and, where applicable, how potential secondary effects (e.g. second site T-DNA insertions, mosiacism, off-target gene editing) were examined.*
